# Trajectories of ethnic discrimination and school adjustment of ethnically minoritized adolescents: The role of school diversity climate

**DOI:** 10.1111/cdev.14133

**Published:** 2024-08-11

**Authors:** Gülseli Baysu, Eva Grew, Jessie Hillekens, Karen Phalet

**Affiliations:** ^1^ Queen's University of Belfast Belfast UK; ^2^ University of Leuven Leuven Belgium; ^3^ Tilburg University Tilburg The Netherlands

## Abstract

This study investigated trajectories of ethnic discrimination experiences in school, diversity climates as contextual antecedents, and school adjustment as outcome. Latent‐Growth‐Mixture‐Models of repeated self‐reported discrimination over 3 years (2012–2015) by 1445 ethnically‐minoritized adolescents of Turkish and Moroccan background in 70 Belgian schools (52.6% boys, *M*_age_ = 15.07) revealed four trajectories: low (72.5%), moderate (16.6%), initially‐high (6.5%), or increasingly high discrimination (4.4%). Adolescents who attended schools with more minoritized peers, or schools that valued cultural diversity and equality, were more often in low‐discrimination trajectories, which predicted better academic outcomes. Overall, school diversity climates can protect minoritized adolescents from experiencing persistent or initially high discrimination over time. Moreover, high discrimination at any point in schooling—initially or later—is harmful to adolescents' school adjustment.

**Abbreviations**
AIC: Akaike information criterion
BIC: Bayesian information criterion
BLRT: bootstrapped likelihood ratio test
CFA: confirmatory factor analysis
FIML: full information maximum likelihood
LGMM: latent growth mixture modeling
LL: loglikehood
LMR‐LRT: Lo–Mendell–Rubin adjusted likelihood ratio test
MAR: missing at random
SES: socioeconomic status

In today's diverse schools, ethnically minoritized adolescents are vulnerable to social exclusion due to their minoritized group status in interethnic relations with teachers and peers (Killen et al., 2013). Ethnic discrimination refers to situations in which ethnically minoritized adolescents perceive themselves as targets of bias, unfair treatment, or hostility due to their background (Civitillo et al., 2024). Such experiences can cast a long shadow over future developmental outcomes, as evident from robust associations with poor school engagement and performance (Benner et al., 2018; Civitillo et al., 2024; Levy et al., 2016). Given persistent ethnic gaps in school outcomes often to the disadvantage of ethnically minoritized youth (Heath & Brinbaum, 2014), it is crucial to understand how ethnic discrimination experiences evolve over time, how they are related to the school environment, and how they predict school adjustment.

Adolescents' ethnic discrimination experiences in school are not always stable but might change for better or worse over time (Benner & Graham, 2011; Greene et al., 2006; Hughes et al., 2016). Moreover, the direction of such changes can differ across adolescents (e.g., Lee et al., 2020; Niwa et al., 2014). Our aim is thus to trace the trajectories of such changes, looking beyond frequencies of discrimination at one point in time or in retrospect or the average change in experienced discrimination over time. Arguably, cumulative, or prolonged experiences of discrimination can be more harmful than momentary experiences (Ladd et al., 2008); however, few studies have traced adolescents' experiences of discrimination over a longer period. Therefore, our *first objective* was to estimate individual trajectories of within‐person change in school‐based ethnic discrimination during adolescence over 3 years. To this end, we employed latent growth mixture modeling (LGMM), a person‐centered approach. Person‐centered approaches seek to identify distinct subgroups or patterns of development within a heterogeneous population (Jung & Wickrama, 2008). In this way, we seek to elucidate the ill‐understood dynamic nature of chronic or transient, initially high or emergent experiences of ethnic discrimination in adolescents' relations with school teachers and peers, which are known to be influential in adolescence (Verkuyten et al., 2019).

We focus on ethnically minoritized youth of Turkish and Moroccan backgrounds as major immigrant communities in Europe. Most of them are the offspring of immigrant workers. They perform worse than their majority peers in school, even when taking into account individual differences and socio‐economic background (Heath & Brinbaum, 2014). Also, they face pervasive prejudice and discrimination as targets of anti‐Muslim sentiment in Europe (Strabac & Listhaug, 2008). We do not know, however, how individual trajectories of discrimination experiences unfold through their school careers (Baysu et al., 2018). Our study thus takes a dynamic approach to evolving discrimination experiences through adolescence. By tracing the discrimination experiences of minoritized youth of Turkish and Moroccan background in Belgian schools, we aim to center perspectives from less researched immigrant communities and migration contexts. With U.S.‐based minoritized youth samples dominating discrimination research (Benner et al., 2018), we newly map individual pathways of school‐based ethnic discrimination among European ethnically minoritized adolescents.

Moreover, we investigate how discrimination trajectories are grounded in the school environment as a critical social and developmental context during adolescence (Eccles & Roeser, 2011). We ask how school diversity climates, i.e., what schools communicate about ethnic diversity, relate to the evolving discrimination experiences of ethnically minoritized adolescents in their relations with teachers and peers. Our *second objective* was thus to predict distinct classes of ethnic discrimination trajectories from aspects of the ethnic diversity climate in schools. We estimated individual trajectories across school contexts with varying levels of ethnically minoritized student presence: from low (under 30%) over medium (30%–60%) to high presence (over 60%). Looking beyond ethnic composition, we assessed student perceptions of school diversity climates. In addition to individual perceptions of minoritized students, we aggregated perceptions of all (minoritized and majority) students in the same school as contextual climate measures. We assessed two possible protective aspects of ethnic diversity climates: perceived equality, i.e., whether all students are treated equally, and multiculturalism, i.e., whether schools value ethnic diversity and act against ethnic discrimination (Baysu et al., 2016, 2021; Heikamp et al., 2020). Accordingly, adolescents who viewed the school diversity climate more negatively experienced more discrimination cross‐sectionally (Benner & Graham, 2013) and over time (Bellmore et al., 2012). Our study newly examines how perceived school diversity climates relate to individual discrimination trajectories to identify possible protective factors in school. Our study adds to literature by newly establishing how distinct trajectories of discrimination among ethnically minoritized youth in Europe are grounded in actual ethnic diversity (ethnic school composition) and perceived ethnic diversity climates, considering both individual and school‐level variation.

Finally, a key question is how discrimination trajectories are related to long‐term school adjustment for minoritized youth in Europe. Against the background of cumulative ethnic disadvantage throughout the school careers of ethnically minoritized youth in European education (Baysu et al., 2018), our *third objective* was to examine the consequences of distinct ethnic discrimination trajectories for their later school adjustment. Extensive research on both sides of the Atlantic associated experiences of ethnic discrimination with lower school adjustment of minoritized youth within and over time (Baysu et al., 2014, 2016; Benner et al., 2018; Benner & Graham, 2013; Brown & Chu, 2012; Verkuyten et al., 2019). However, few studies focused on the long‐term adjustment consequences of distinct individual discrimination trajectories during adolescence. As research connecting discrimination trajectories with school outcomes is still limited (Niwa et al., 2014), we do not know the precise developmental impact of persistent, initially high, or emerging discrimination experiences. Our study will hence explore how distinct classes of individual trajectories interrelate with changes in various affective and behavioral aspects of school adjustment (i.e., school belonging, (dis)engagement, achievement, and (non)compliance).

## Trajectories of ethnic discrimination experiences in school

Adolescence is a critical developmental period when peer relations in school gain importance (Eccles & Roeser, 2011). It is also a period when young people's views on ethnic diversity and interethnic relations are formed (Killen et al., 2013). As ethnically or racially minoritized adolescents are coming to terms with their minoritized group status in interethnic relations, they become more aware of and vulnerable to discrimination (Baysu et al., 2016). There are a few longitudinal studies of minoritized adolescents' ethnic discrimination experiences; and their findings were mixed, showing increasing (Benner & Graham, 2011; Brody et al., 2006; Greene et al., 2006; Hughes et al., 2016), decreasing (Bellmore et al., 2012; Hughes et al., 2016), or stable average trends over time (Greene et al., 2006). Mixed findings might reflect heterogeneity in adolescents' experiences of discrimination over time, as suggested by significant variability around average trends in some studies (Bellmore et al., 2012; Brody et al., 2006; Greene et al., 2006; Hughes et al., 2016). Unlike longitudinal methods that assume a single homogeneous trajectory, LGMM allows for the exploration of different discrimination trajectories in the data. This person‐centered approach is valuable when studying complex phenomena that may exhibit diverse developmental patterns between individual persons or hidden subpopulations (Jung & Wickrama, 2008). Therefore, our study looks for individual differences in minoritized adolescents' discrimination trajectories.

Moving beyond general developmental trends, we know of five longitudinal studies that modeled different classes of ethnic discrimination trajectories using LGMM. All five studies focused on U.S.‐based ethnically or racially minoritized samples (mostly African American and Latinx), ranging in age from early adolescence to emerging adulthood (Lee et al., 2020; Niwa et al., 2014; Smith‐Bynum et al., 2014; Tynes et al., 2020; Unger et al., 2016). Four common types of individual trajectories emerged across these five studies. Low levels of discrimination were generally the most common, as evident from low‐stable and low‐decreasing trajectories of discrimination (Lee et al., 2020; Niwa et al., 2014; Smith‐Bynum et al., 2014; Unger et al., 2016). Besides low levels of discrimination, many minoritized adolescents followed pathways with *some* discrimination experiences either initially or over time. For some adolescents, initially negative yet improving interethnic experiences were indicated by high‐ or moderate‐decreasing trajectories of discrimination (Lee et al., 2020; Niwa et al., 2014; Smith‐Bynum et al., 2014; Tynes et al., 2020; Unger et al., 2016). For others, discrimination worsened over time as evident from low‐ or moderate‐increasing trajectories of discrimination (Lee et al., 2020; Smith‐Bynum et al., 2014; Tynes et al., 2020; Unger et al., 2016). In all five studies, some adolescents experienced moderate‐ or high‐stable trajectories of discrimination. Most studies (except for Niwa et al., 2014) focused on discrimination experiences in general, rather than specifically within the school.

Building on these studies, we used repeated measures of school‐based ethnic discrimination among ethnically minoritized adolescents of Turkish and Moroccan background to replicate different individual trajectories of discrimination in Belgian secondary schools. We expected that most ethnically minoritized adolescents would experience little discrimination (low‐stable or low‐decreasing trajectories; Niwa et al., 2014; Smith‐Bynum et al., 2014; Unger et al., 2016). Besides, we expected smaller numbers to face more severe discrimination either initially (high‐ or moderate‐decreasing trajectories; Lee et al., 2020; Niwa et al., 2014; Smith‐Bynum et al., 2014; Tynes et al., 2020; Unger et al., 2016), or increasingly (low‐ or moderate‐increasing trajectories; Lee et al., 2020; Smith‐Bynum et al., 2014; Tynes et al., 2020; Unger et al., 2016), or persistently over time (high‐ or moderate‐stable trajectories; Lee et al., 2020; Niwa et al., 2014; Smith‐Bynum et al., 2014; Tynes et al., 2020; Unger et al., 2016). Given evidence associating school‐based discrimination with adjustment problems (Verkuyten et al., 2019), we focused on school‐based discrimination trajectories as opposed to more general discrimination experiences used in previous studies.

## School diversity climates as antecedents of ethnic discrimination

Positive school diversity climates may protect ethnically minoritized adolescents through buffering (i.e., reducing) their discrimination experiences. To explain how school diversity climates shape discriminatory experiences, we bridge an ecological approach to ethnically minoritized adolescents' development (Syed et al., 2018) with a social identity approach to their intergroup relations with majority teachers and peers in school (Phalet & Baysu, 2020). Drawing on an ecological approach (Bronfenbrenner & Morris, 2006), we view school as a critical microsystem that shapes adolescents' development and school adjustment through social interactions with peers and teachers. Our explanatory focus is on diversity climates in today's ethnically diverse educational settings. The term “school diversity climate” refers to how schools and teachers recognize, value, and manage ethnic diversity through various policies and practices (Baysu et al., 2021; Wang & Degol, 2016). As a critical protective factor in ethnically minoritized adolescents' school environment, diversity climates afford more or less adaptive school trajectories and outcomes through affecting the quality of interactions experienced by these adolescents (Celeste et al., 2019; Thapa et al., 2013; Wang & Degol, 2016). While school‐based interactions can be positive such as instances of positive contact with peers or teachers, our study aims to advance our understanding of negative interactions, particularly instances of peer or teacher discrimination (Baysu et al., 2016, 2021). Centering the school experiences of ethnically minoritized youth, we draw on a social identity approach to intergroup relations in the school context to articulate the processes that connect school diversity climates to minoritized adolescents' experiences of ethnic discrimination (Phalet & Baysu, 2020; Verkuyten et al., 2019). From a social identity approach, positive school diversity climates can create an identity‐safe environment where ethnically minoritized adolescents feel that their minoritized identity is valued and that all students are treated equally (Phalet & Baysu, 2020). In such an environment, minoritized adolescents should experience less discrimination. Below we theorize identity‐related processes about two potential protective factors in perceived school diversity climates.

These potential protective factors are whether students see their school as ensuring fairness or equal treatment of all students (i.e., an equality climate; Baysu et al., 2016); and whether they see their school as valuing ethnic (or religious) differences and acting against ethnic discrimination (i.e., a multicultural climate; Heikamp et al., 2020). As research on school climates has been largely restricted to individual perceptions (Wang & Degol, 2016) as predictors of discrimination (Benner & Graham, 2011, 2013; Brown & Chu, 2012), our study extends previous findings by defining school climates primarily at the collective level as shared perceptions among all students. Our study thus supplements minoritized adolescents' individual perceptions with aggregated perceptions across minoritized and majority students within the same school as more contextual measures of diversity climates. We also assessed objective levels of ethnically minoritized student presence in schools to consider actual levels and patterns of ethnic diversity across schools. Indeed, the presence of minoritized peers was revealed as buffering against discrimination (Kende et al., 2021).

How would individual and shared perceptions of a positive school diversity climate protect ethnically minoritized students from frequent or prolonged ethnic discrimination experiences? First, the equality component of a protective diversity climate should protect minoritized adolescents from discrimination, through endorsing and communicating fairness as a developmentally salient concern (Killen et al., 2013). Accordingly, for minoritized youth, perceived fairness is associated with positive psychological and school adjustment (Baysu et al., 2016; Benner & Graham, 2011; Schachner et al., 2019), higher quality relationships with their teachers (Baysu et al., 2021), and less discrimination (Benner & Graham, 2011, 2013; Juvonen et al., 2018). *We thus expected that in schools where ethnically minoritized students perceived more fairness at time 1, low‐discrimination trajectories would be more likely over time (relative to other discrimination trajectories)*. We also explored a possible school‐level effect of shared perceptions of fairness over and above individual perceptions.

Second, a multicultural school climate (Benner & Graham, 2013) may also protect ethnically minoritized adolescents from discrimination, by valuing their ethnic or religious identities and communities and acting against disrespect and ethnic discrimination (Verkuyten et al., 2019). Accordingly, when schools and teachers valued diversity, minoritized students had higher school achievement and adjustment (Celeste et al., 2019; Schachner et al., 2019) and reported less discrimination cross‐sectionally (Benner & Graham, 2013; Brown & Chu, 2012) and longitudinally (Bellmore et al., 2012). *We thus expected that in schools where ethnically minoritized students perceived more multiculturalism at time 1, low‐discrimination trajectories (vs. other trajectories) would be more likely*. We again explored a possible school‐level effect of shared perceptions of multiculturalism.

We also explored whether schools with a higher presence of ethnically minoritized peers as possible allies in interethnic relations might be an alternate source of protection from frequent or prolonged discrimination experiences. We focused on school ethnic composition which refers to the proportion of ethnically minoritized versus majority adolescents in a school. Previous findings on discrimination and ethnic composition are mixed, relating higher shares of minoritized students in school to either increased (Benner & Graham, 2011; Brown & Chu, 2012) or decreased experiences of discrimination and exclusion (Agirdag et al., 2011; Juvonen et al., 2018). Still, others found a curvilinear association that self‐reported discrimination increases when minoritized student presence goes up and drops off again at very high levels when minoritized students outnumber majority students (Baysu et al., 2014; Bellmore et al., 2012). Accordingly, *we explored whether low (vs. other) discrimination trajectories over time would be most likely in schools with low (<30%), medium (30%–60%), or high levels (>60%) of ethnically minoritized student presence at time 1*.

## Changes in school adjustment as consequences of ethnic discrimination

To explain the developmental consequences of discrimination for minoritized adolescents' school adjustment, we again start from an ecological approach to their development (García Coll et al., 1996; Syed et al., 2018). Accordingly, race or ethnicity and related experiences such as discrimination are essential for an accurate understanding of ethnically minoritized adolescents' developmental trajectories and outcomes. Adding on a social identity approach, we theorize underlying identity‐related processes so that experiences of ethnic discrimination pose a direct threat to minoritized adolescents' salient group identity and fairness concerns (Killen et al., 2013). In turn, adolescents can reduce their efforts and disengage from the domains that induce these threats (Major & O'Brien, 2005). Applying this to the school context, ethnically minoritized adolescents can thus respond by distancing themselves behaviorally and psychologically from school as a coping mechanism (Verkuyten et al., 2019), which can be a costly strategy for various aspects of their school adjustment.

At the empirical level, recent meta‐analyses (Benner et al., 2018; Civitillo et al., 2024; Verkuyten et al., 2019) converge on the key role of discrimination in the development of ethnically minoritized adolescents, with possible adverse impact on their school adjustment. Cross‐sectional studies related discrimination experiences in school to disengagement from learning and underachievement (Baysu et al., 2014, 2016). Longitudinal studies on average trends found that initial experiences of ethnic discrimination, as well as increases in discrimination over time, were associated with worse developmental outcomes (Benner & Graham, 2011; Hughes et al., 2016). Others exploring the bi‐directionality of associations between discrimination and adjustment found that while earlier experiences of discrimination predicted later adjustment, the reverse was not true (Cheng et al., 2020).

Despite converging cross‐sectional and longitudinal evidence on the developmental costs of ethnic discrimination for school outcomes of minoritized adolescents (Benner et al., 2018), we do not know whether chronic, initially high, or emergent experiences of discrimination are differentially associated with worse school outcomes. Either early or emergent discrimination experiences can put minoritized students at risk, especially around critical transitions in school careers that can impact their future life chances (Baysu et al., 2018). Only one study that we know of related ethnic discrimination trajectories to school adjustment in the U.S. (Niwa et al., 2014). They found that adolescents who experienced high‐decreasing discrimination from peers in school felt less school belonging, while those who experienced moderate levels of discrimination did not differ from those who reported no discrimination. In this study, initially high levels of early discrimination were more detrimental than prolonged moderate levels of discrimination, yet we need more research to arrive at any conclusions. We thus expected that *ethnically minoritized adolescents in low‐discrimination trajectories would show better school adjustment over time (compared to other trajectories)* and explored the differential consequences of other discrimination trajectories.

## Current study

This study focuses on ethnically minoritized groups of Turkish and Moroccan background as the largest minoritized groups in Belgium originating from non‐EU nations. Although Belgium lacks official ethnicity statistics, it is estimated that about 2% have Turkish heritage and 3.8% have Moroccan ancestry (Galle et al., 2020). Most of them are descendants of immigrant workers, originating in post‐1960s labor migration, from Turkey and Morocco as majority Muslim countries. While many hold Belgian citizenship, this does not equate to equal status. They face disparities in education, employment, and income (Heath & Brinbaum, 2014) and face discrimination, as targets of anti‐Muslim sentiment in Europe (Strabac & Listhaug, 2008). Their dual position—legal insiders, yet societal outsiders—may intensify their experiences of discrimination (Alanya et al., 2015; Galle et al., 2020). This context underscores the significance of investigating perceived discrimination in this group.

Moreover, Belgian secondary schools present additional structural challenges for minoritized students. Hierarchical tracking systems stream students into vocational, technical, or academic tracks based on their early performance. Minoritized students, even when achieving early academic success, are disproportionately directed toward vocational education compared to majority students (Baysu et al., 2018). These decisions are often irreversible and determine students' future opportunities (Baysu et al., 2018; Eccles & Roeser, 2011). Thus, it is crucial to study experiences of ethnic discrimination within school settings.

Finally, Europe has been critiqued by multiple scholars for avoiding discussions about race, despite the pervasive presence of embedded racism (Jugert et al., 2022; Moffitt et al., 2020). In continental Europe, researchers and policymakers often adopt the term “migration‐background” to encompass ethnically minoritized groups based on one's own place of birth and that of one's parents and grandparents. We similarly use parentage to define the ethnically minoritized groups of immigrant background in this study. Moreover, in our study, we do not explicitly refer to “ethnic” or “racial” discrimination in our discrimination measure due to the distinct context of our research. In public discourse, these terms may not commonly appear (Jugert et al., 2022; Moffitt et al., 2020), and we aimed to ensure that our survey questions resonated with the experiences and language familiar to our participants. Instead, our discrimination measure asks participants about their perceptions regarding the basis of their encountered discrimination in school. This approach enables us to understand how participants perceive the underlying factors of discrimination and to what extent these factors can be interpreted through an ethnic or racial lens.

In summary, drawing on large‐scale school‐based longitudinal data (three cohorts, three waves) with ethnically minoritized youth of Turkish and Moroccan background in a European migration context, our study aimed to elucidate trajectories of within‐person change or stability in their school‐based ethnic discrimination experiences, their social grounding in school diversity climates as contextual antecedents, and their long‐term consequences for changes in school adjustment as key developmental outcomes for minoritized youth.

## METHOD

### Participants

The data were part of a large‐scale longitudinal survey with three annual waves in 70 secondary schools in Flanders, Belgium (Children of Immigrants Longitudinal Survey Belgium, 2012–2015; Phalet et al., 2018). After obtaining consent from respective parties in line with the university ethical guidelines, students filled out a questionnaire in class in the presence of research assistants. Schools were stratified based on schools' administrative information about the proportion of students who spoke foreign languages at home, varying from low (<30%) through moderate (30%–60%) to high stratum (above 60%). Participants were randomly selected from either the first (30.1%), second (31.9%), or third (38.0%) year of secondary education (i.e., similar to U.S middle school; normative ages 12–15 years). We focused on minoritized adolescents of Turkish (44.4%) and Moroccan background (55.6%). Those who were either born in Turkey or Morocco or had a parent or grandparent born there were included in the sample (based on adolescents' self‐report). For school‐level aggregated perceptions of school diversity climates, we also included the reports of 1875 Belgian adolescents who were born in Belgium and had parents and grandparents born in Belgium too.

Minoritized adolescents (*N* = 1445; 47% girls, 53% boys) were on average 15.07 years old at time 1 (SD = 1.23, range: 12.62 to 19.85 years; 76% less than 16 years old), mostly Muslims (94.1%) and second‐generation, indicating that the adolescent was born in Belgium while (one of) the parents were born in Turkey or Morocco (77%). The Belgian educational system has a hierarchical tracking structure that streams students into different school tracks for vocational, technical, or academic secondary education at an early age. Academic tracks are more selective and geared toward higher education, technical tracks prepare for higher education and work, whereas vocational tracks lead directly to the job market (Baysu et al., 2018; Eccles & Roeser, 2011). Half of our sample enrolled in vocational school tracks (48.7%, vs. 31.6% academic, or 19.7% technical). In terms of parental education as a proxy for socioeconomic status (SES), only 19% had parents who had higher education (vs. secondary school or lower).

Sample selection was not determined by power analysis. The sampling strategy involved selecting more students from the high stratum (schools with 60% or more students with a migrant background), ensuring the inclusion of a sufficient count of students with migrant backgrounds in the sample. Accordingly, the aim was to recruit approximately 40 pupils in schools with low‐to‐moderate ethnic strata (<60%) and 60–70 students in schools with the highest stratum (>60%), with a total of 1500 ethnically minoritized students with an immigrant background from as many schools as possible (Phalet et al., 2018).

### Measures

To indicate at which waves (or times) the variables were measured, from here onward we used T1, T2, and T3 referring to Time 1, Time 2, and Time 3, each approximately 1 year apart between 2012 and 2015.

*Experiences of ethnic discrimination (T1, T2, T3)* were measured with one item in each wave to identify discrimination trajectories (Baysu et al., 2016): “How often are you being discriminated against, treated unfairly, or with hostility in school?” (from 1 = *Never* to 4 = *(Almost) always*). Although the question did not refer to ethnic background per se, those who faced some discrimination at T1 (scoring two or above, 24%) were asked to tick all the reasons for their experiences of discrimination. The most frequent reasons adolescents mentioned indicated ethnically‐motivated discrimination: “another religion” (11%), “another language at home” (7.5%), “another country” (7.1%), and “skin color” (4.3%). Other reasons like “another neighborhood” (6.8%) and “the way they look” (5.6%) could indicate ethnic differences as well since the ethnically minoritized often live in segregated and poor neighborhoods in Belgium (Costa & De Valk, 2018), and the way they look could relate to racial or religious differences. Two additional categories were “gender” (2.6%) and “other reasons not listed” (6.6%). Minoritized adolescents were also asked about the sources of discrimination: peers (10.8%), teachers (8.7%), and school administration or other school personnel (6.8%). Since participants were allowed to choose more than one reason or source, these are not cumulative percentages. Section S1 outlines the re‐coding and description of these variables and descriptive analyses.

The following measures of perceived school diversity climate were administered at T1, utilizing a scale from 1 = *Strongly disagree* to 5 = *Strongly agree*:

*Perceived equality in school (T1)* was measured with two items: “The rules are applied equally to all students” and “Some students are allowed more than others” (reverse‐coded). Items were derived from the Experience of School Rules scale (Gregory et al., 2011) and used in previous research (Baysu et al., 2016, 2021). We used both individual‐level adolescent perceptions (Spearman‐Brown *α* = .53) and aggregated (i.e., averaged) perceptions of minoritized adolescents of Turkish and Moroccan background and majority adolescents within the same school as a school‐level variable (Spearman‐Brown *α* = .61).

*Perceived multiculturalism in school (T1)* was measured with four items such as “In my school, different cultures and religions are treated with respect” or “In my school, they take strong action against racism and discrimination.” Items were derived from the Teachers' Multicultural Attitudes Scale (Green et al., 1988; Thijs et al., 2012) and used in previous research (Baysu et al., 2021). We used both individual‐level adolescent perceptions (*α* = .67) and school‐level aggregated perceptions (*α* = .68).

*Objective levels of ethnic school composition (T1)* were based on schools' administrative information about the proportions of students who spoke foreign languages at home. We differentiated between low (<30% as the reference), moderate (30%–60%), and high (>60%) shares of ethnically minoritized students using dummy coding at the school level.

The following school outcomes were measured at T3, controlling for scores at T1.

*Achievement (T1, T3)* referred to self‐reported math and Dutch grades. Since schools use different grading systems, we re‐coded their grades to a scale from 0–100 to make them comparable across schools (with higher scores indicating higher achievement).

*School engagement (T1, T3)* distinguished between emotional engagement (e.g., “I like to be in class”; *α*_T1_ = .74, *α*_T3_ = .70), behavioral engagement (e.g., “I work as hard as I can in class”; *α*_T1_ = .81, *α*_T3_ = .81), and behavioral disaffection (e.g., “I often think of other things during class”; *α*_T1_ = .62, *α*_T3_ = .59). Each dimension was measured with three items adapted from the Engagement versus Disaffection with Learning scale (Skinner et al., 2008), utilizing a 5‐point scale ranging from 1 = *Strongly disagree* to 5 = *Strongly agree*. This scale was also used in previous studies (Baysu et al., 2021; Hillekens et al., 2023).

*School belonging (T1, T3)* was measured with four items (Wang et al., 2011), such as “I feel at home at this school” or “I am proud to be a pupil of this school” (*α*_T1_ = .85, *α*_T3_ = .84) on a scale from 1 = *Strongly disagree* to 5 = *Strongly agree*.

*School non‐compliance (T1, T3)* was measured with three items: “getting punishment in school,” “skipping a lesson without permission,” and “coming late to school” (from 1 = *Never* to 5 = *Every day*; *α*_T1_ = .56, *α*_T3_ = .55; Wang et al., 2011). This scale was also used in previous studies (Baysu et al., 2021).

*Control variables (T1)* were age (T1), gender (girls vs. boys as reference), school track (vocational vs. non‐vocational tracks as reference), and parental education as a proxy for SES (higher education vs. secondary or lower as reference). Turkish (vs. Moroccan) background was dropped from the analyses as it had no significant effects.

### Analytic strategy

Using Mplus 8.3 (Muthén & Muthén, 1998–2017), we performed a confirmatory factor analysis (CFA) to establish the psychometric properties of all study measures and a multigroup CFA across Turkish and Moroccan‐background minoritized groups. These analyses (presented in Section S2) confirmed the validity of our measures. The main analyses were then conducted in four parts. First, using LGMMs we identified different trajectories of ethnic discrimination. Second, adolescents were assigned to their most likely trajectory based on posterior probabilities. Using multilevel multinomial regression, these trajectories (as dependent variables) were predicted by perceived equality and multiculturalism (both at the individual and school level), and objective ethnic school composition (at the school level). Third, we performed separate multilevel regression analyses for each school outcome at time 3 (i.e., controlling for T1 outcome; grades, non‐compliance, engagement, and belonging) with the trajectories as dummy‐coded predictors. Controlling T1 outcomes allows for predicting the *change* in outcomes over time. Missing data were handled using full information maximum likelihood estimation (FIML) which uses all available data without imputing missing data and is thus unbiased and preferable to other methods (Dong & Peng, 2013). Details on model specifications can be found in Section S3.

### Transparency and openness

We reported all the measures, analyses, and data exclusions transparently (see Supporting Information for additional information about all the models and Phalet et al., 2018 for the technical report and the questionnaire). This study's design and its analysis were not pre‐registered. However, all the Mplus syntax and outputs and the data that we used are available on the OSF page, link. As this is a large‐scale longitudinal data unique with its focus and measures in Europe, it was partially used in other publications, but the current study is the only one analyzing repeated experiences of ethnic discrimination over 3 years. No published studies we know have used these data to address research questions related to trajectories of discrimination, its antecedents, or longitudinal consequences (For a comparison with the past publications using this data, see Section S4). This study was mainly confirmatory, yet the examination of the number and types of trajectories remained exploratory due to the nature of the analyses. The analysis of differential consequences of discrimination trajectories, beyond high and low, was also exploratory.

## RESULTS

Descriptive statistics are reported in Table 1.

**TABLE 1:** Descriptive statistics.

	M/%	SD	1	2	3	4	5	6	7	8	9	10	11	12	13	14	15	16	17	18	19	20	21	22	23	24	25	26
School‐level variables																												
1. Moderate school composition (30%–60%)	35%																											
2. High school composition (>60%)	21%		−.72***																									
3. T1 perceived equality	3.55	0.27	−.26***	.29***																								
4. T1 perceived multiculturalism	3.79	0.22	−.30***	.23***	.63***																							
Individual‐level variables																												
5. T1 perceived equality	3.57	1.02	−.04	.06*	.24***	.17***																						
6. T1 perceived multiculturalism	3.76	0.85	−.04	.10***	.12***	.20***	.20***																					
7. T1 discrimination	1.33	0.68	.02	−.05	−.09**	−.06*	−.15***	−.12***																				
8. T2 discrimination	1.39	0.71	.01	−.03	−.06	−.04	−.15***	−.09**	.30***																			
9. T3 discrimination	1.43	0.79	.08*	−.08	−.11**	−.07	.38***	.19***	.19***	.41***																		
10. T1 school belonging	3.46	0.98	−.01	−.01	.24***	.19***	.26***	.12**	−.16***	−.13***	−.08																	
11. T3 school belonging	3.45	0.94	−.03	.06	.11**	.07	−.19***	−.02	−.09*	−.20***	−.22***	.37***																
12. T1 school non‐compliance	1.66	0.59	.01	.01	−.18***	−.13***	−.18***	−.04	.10***	.07*	.04	−.24***	−.07															
13. T3 school non‐compliance	1.68	0.57	.03	.02	−.12**	−.06	.01	.03	.16***	.10*	.16***	−.13***	−.17***	.45***														
14. T1 math grades	57.22	24.40	−.05	−.04	−.10*	.05	0	.07	−.05	0	−.04	.02	.09	.02	−.01													
15. T3 math grades	66.04	13.63	−.01	.01	−.05	−.07	.01	.02	−.02	.02	−.02	−.09*	.01	−.08	−.19***	.15*												
16. T1 Dutch grades	57.14	23.44	−.04	−.06	−.09*	.08*	−.02	.03	.04	.04	.01	−.01	.02	.02	.03	.81***	.08											
17. T3 Dutch grades	66.78	12.05	.01	−.01	−.03	−.05	.19***	.09***	.01	0	.01	−.11*	−.04	−.08	−.19***	.02	.57***	.14*										
18. T1 behavioral engagement	3.79	0.69	−.06*	.10***	.05	.01	.07	−.02	−.01	−.04	.03	.27***	.09*	−.33***	−.17***	.12***	.14***	.08	.12***									
19. T3 behavioral engagement	3.66	0.67	.01	.01	−.06	−.07	−.23***	−.02	−.01	.02	0	.05	.22***	−.12***	−.22***	.13*	.19***	.10	.18***	.40***								
20. T1 behavioral disaffection	2.75	0.85	.08***	−.09***	−.13***	−.10***	−.10*	.06	.10***	.10**	.03	−.23***	−.12***	.35***	.23***	−.06	−.15***	.01	−.13***	−.45***	−.31***							
21. T3 behavioral disaffection	2.91	0.79	−.03	.01	.00	.05	.30***	.18***	.10*	.06	.12**	−.05	−.20***	.16***	.35***	−.02	−.20***	−.03	−.19***	−.24***	−.40***	.38***						
22. T1 emotional engagement	3.84	0.81	−.03	.08***	.11***	.06*	.14**	.07	−.13***	−.09**	−.05	.52***	.27***	−.25***	−.14***	.06	.07	.03	.02	.46***	.24***	−.27***	−.15***					
23. T3 emotional engagement	3.74	0.69	.01	.04	−.06	−.07	.38***	.19***	−.01	−.10*	−.12**	.20***	.53***	−.07	−.14***	−.03	.06	−.04	.02	.16***	.47***	−.15***	−.16***	.36***				
24. Age	15.07	1.23	.03	−.02	−.13**	−.19***	−.10***	−.07*	.02	.04	−.06	−.17***	−.01	.16***	−.02	.06	.05	.03	.00	−.07**	.06	.08***	.00	−.08***	.04			
25. School track: vocational	49%		.01	.16***	−.22***	−.26***	−.05	−.06*	.07*	.04	.04	−.07**	.00	.15***	.06	.08	.23***	.01	.20***	.08***	.12***	−.01	−.05	.11***	.11**	.09***		
26. Gender: female	47%		.10***	−.08***	.02	.06*	.04	−.07*	−.08**	−.09**	−.09*	.10***	.03	−.11***	−.15***	.01	−.07	.05	.04	−.01	−.03	−.03	−.01	.05	−.06	.03	−.02	
27. Parental education: higher	19%		−.03	−.02	.00	.04	−.02	.01	.09**	.01	.06	.01	.01	.04	.04	−.07	.02	−.04	.02	−.02	.01	.08***	.05	.03	.05	−.08**	−.08*	−.10***

* *p* < .05;

** *p* < .01;

*** *p* < .001.

### Attrition analyses

We compared adolescents who completed all three waves (40%) to those who missed at least one wave of the data collection in all the study variables (i.e., 23% completed only T1 and T2, 6% completed only T1 and T3, and 31% of adolescents completed only T1). Those who missed at least one wave reported more discrimination at T1, Welch's *F*(1, 1287.32) = 15.31, *p* < .001. They also reported worse school outcomes: lower school belonging, Welch's *F*(1, 1308.70) = 40.95, *p* < .001, more non‐compliance, *F*(1, 1406) = 21.85, *p* < .001, lower grades in both Math, Welch's *F*(1, 493.09) = 13.24, *p* < .001, and Dutch at T1, *F*(1, 494.71) = 8.47, *p* = .002, and lower behavioral, Welch's *F*(1, 1311.66) = 19.50, *p* < .001, and emotional engagement, *F*(1, 1409) = 13.72, *p* < .001, and higher behavioral disaffection, *F*(1, 1409) = 19.14, *p* < .001. Although Little's test was not significant (*χ* ^2^(1599) = 1663.56, *p* = .127), suggesting support for missing completely at random assumption, the differences in observed variables are more in line with the missing at random (MAR) assumption where missingness can depend on the previous history of the variables or other observed variables, for which the FIML method we used was found to be robust (see Section S5).

### Trajectories of ethnic discrimination

We first ran a latent growth curve model to estimate the average intercept and slope of discrimination (see Section S6). While on average discrimination was slightly increasing over time (*I* = 1.35, *S* = 0.06, *p* = .009), significant variation around the intercept and slope suggested the presence of latent subgroups. After running one to five trajectory models, we chose the four‐trajectory model as our final model (see Table 2 for the model fit indices and Section S7 for details). The final model choice in LGMM depends on the model fit indicators and the theoretical significance of each trajectory (Jung & Wickrama, 2008; Nylund‐Gibson & Choi, 2018). Comparing the three to four trajectory models, a four‐trajectory model provided better‐fit statistics: Loglikehood (LL), the Akaike and Bayesian information criterion (including sample adjusted) (AIC, BIC) yielded lower values, and likelihood ratio tests (Lo–Mendell–Rubin adjusted likelihood ratio test‐LMR‐LRT and the bootstrapped likelihood ratio test‐BLRT) showed a significant improvement. In the four‐trajectory model, the additional trajectory (with an increasing trend) was a large enough group (4.4%) and of theoretical value. Comparing the four to five trajectory models, while LMR‐LRT and BLRT suggested significant improvement, LL, AIC, and BIC suggested little improvement. The fifth class did not add much theoretical value: it was a small group (1.4% or 20 people) of a similar increasing trajectory. We established the four‐trajectory solution as the final model (Section S7).

**TABLE 2:** Model fit indicators for ethnic discrimination trajectories: 1 to 5 class solutions.

	1 Class	2 Class	3 Class	4 Class	5 Class
Model fit					
LL	−2933.74	−2564.41	−1645.827	−1547.775	−1468.269
AIC	5883.48	5150.82	3319.65	3129.55	2976.54
BIC	5925.37	5208.42	3392.96	3218.57	3081.27
Adj. BIC	5899.96	5173.47	3348.49	3164.57	3017.73
Entropy	1.00	0.98	0.97	0.90	0.90
LMR‐LRT		706.14, *p* < .001	1756.26, *p* = .020	187.47, *p* = .009	152.01, *p* = .001
BLRT		738.67, *p* < .001	1837.16, *p* < .001	196.10, *p* < .001	159.01, *p* < .001
%					
Low	100%	93.7%	77.0%	72.5%	72.7%
High‐decreasing	(—)	6.3%	6.5%	6.5%	6.5%
Moderate	(—)	(—)	16.5%	16.6%	15.0%
Low‐increasing	(—)	(—)	(—)	4.4%	4.3%
Moderate‐increasing					1.4%

*Note*: Trajectories were estimated using linear growth models.

Abbreviations: AIC, Akaike information criterion; BIC/Adj. BIC, Bayesian information criterion/sample size adjusted BIC; BLRT, bootstrapped likelihood ratio test; LL, loglikehood; LMR LRT, Lo–Mendell–Rubin likelihood ratio test.

**FIGURE 1:**
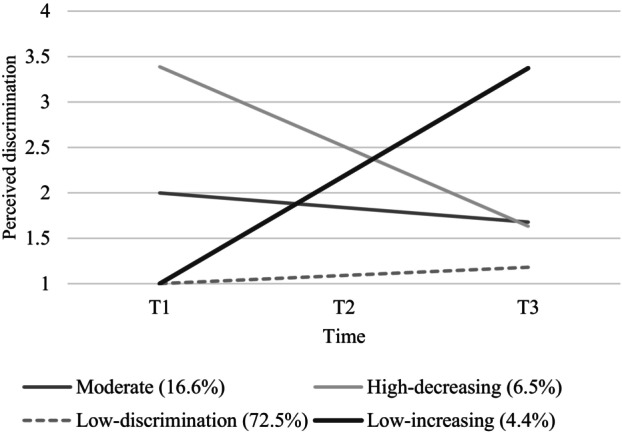
Trajectories based on estimated means of perceived ethnic discrimination at T1, T2, and T3.

As seen in Figure 1, most adolescents (72.5%) followed the low‐discrimination trajectory. Adolescents in this trajectory consistently reported low discrimination (i.e., never discriminated) across all three waves, despite a negligible increase over time (*I* = 1.00, *S =* 0.09, *p* < .001). We also found three trajectories with higher levels of discrimination. In the moderate trajectory (16.6%), adolescents experienced moderate levels of discrimination (i.e., sometimes) that slightly decreased over time (*I* = 2.00, *S =* −0.16, *p* = .001). In the high‐decreasing trajectory (6.5%), adolescents experienced initially high (often‐to‐always) discrimination which decreased over time (*I* = 3.39, *S =* −0.89, *p* < .001). We also found a low‐increasing trajectory (4.4%; *I* = 1.00, *S =* 1.19, *p* < .001), when adolescents reported initially low discrimination, but their discrimination experiences rapidly increased over time.

We ran additional descriptive analyses to tabulate discrimination trajectories with sources of and reasons for discrimination (see Section S1 for details). Most students thought they had been discriminated against for ethnic reasons in both the moderate and the high‐decreasing trajectories. As for the sources of discrimination, in the high‐decreasing trajectory teachers and school personnel were the main sources of discrimination at T1 whereas, in the moderate trajectory, discrimination was equally perpetrated by either peers or teachers and school personnel. As we only had this additional information from T1 and most adolescents in the low‐increasing and low‐stable trajectories reported no discrimination at T1, they were not asked these additional questions. Thus, we could not perform similar comparisons for them.

### School diversity climate predicting ethnic discrimination trajectories

As seen in Table 3, minoritized adolescents who perceived their school as more multicultural were more likely to be in the low‐discrimination trajectory (vs. high‐decreasing or moderate‐discrimination trajectories). Similarly, when minoritized students perceived their school as fairer, they were more likely to be in the low‐discrimination trajectory (vs. the high‐decreasing trajectory). The fairness effect was marginally replicated at the school level with aggregate views of both minoritized and majority students on fairness (*p* = .080). Moreover, in schools with high shares of minoritized students (>60%), adolescents were significantly more likely to be in the low‐discrimination trajectory (vs. the moderate‐discrimination), compared to schools with low (<30%) shares of minoritized students. In sum, perceiving school as a place where students are treated equally and where different cultural backgrounds are valued and being in schools with larger shares of minoritized peers, were associated with consistently reporting low discrimination over time. As for demographics, girls were more likely to be in the low‐discriminatory (vs. the high‐decreasing) trajectory than boys. Those in a vocational school track tended to be less likely to be in the low‐discrimination trajectory (vs. the high‐decreasing and low‐increasing trajectories) than those in the academic tracks.

**TABLE 3:** Multilevel multinomial regression predicting ethnic discrimination trajectories by school diversity climate.

	Moderate		High‐decreasing		Low‐increasing	
	*B* (SE)	OR	*B* (SE)	OR	*B* (SE)	OR
Individual level						
Main effects						
T1 perceived equality	−0.11 (.08)	0.90	−0.43 (.07)***	0.65	−0.14 (.12)	0.87
T1 perceived multiculturalism	−0.47 (.10)***	0.63	−0.31 (.15)*	0.73	−0.31 (.20)	0.73
Controls						
T1 age	−0.02 (.07)	0.98	−0.01 (.09)	0.99	−0.11 (.12)	0.90
School track: vocational	0.22 (.16)	1.24	0.56 (.32)^a^	1.75	0.50 (.27)^a^	1.65
Gender	−0.14 (.16)	0.87	−0.56 (.22)*	0.57	−0.29 (.30)	0.75
Parental education: higher	0.27 (.17)	1.31	0.67 (.27)*	1.96	0.28 (.37)	1.32
School‐level						
Main effects						
T1 perceived equality	0.52 (.38)		−1.42 (.81)^a^		−0.25 (.63)	
T1 perceived multiculturalism	0.41 (.38)		1.09 (.78)		−0.11 (.85)	
Moderate school composition (30%–60%)	−0.08 (.18)		−0.20 (.28)		0.12 (.34)	
High school composition (>60%)	−0.59 (.21)**		−0.25 (.30)		−0.18 (.37)	

*Note*: The table presents unstandardized regression coefficients with standard errors in parenthesis. “Low‐discrimination” trajectory as the reference.

Abbreviation: OR, odds ratios.

^a^ *p* ≤ .08;

* *p* < .05;

** *p* < .01;

*** *p* < .001.

### Ethnic discrimination trajectories predicting school outcomes

Minoritized adolescents in the low‐discrimination trajectory had better school outcomes at T3 than those in high‐discrimination trajectories, controlling for T1 outcomes (Table 4). Minoritized adolescents in the low‐discrimination trajectory reported significantly higher school belonging, and emotional engagement (and slightly more behavioral engagement, *p* = .072) than those in the low‐increasing trajectory. They also reported less non‐compliance in school (vs. high‐decreasing trajectory) and less behavioral disaffection (vs. low‐increasing and high‐decreasing trajectories). Also, older adolescents reported lower behavioral disaffection than younger ones, and girls reported less non‐compliance than boys.

**TABLE 4:** Separate multilevel regression analyses predicting school outcomes at T3 by ethnic discrimination trajectories.

	Belonging model		Non‐compliance model		Engagement model					
	T3 school belonging		T3 school noncompliance		T3 behavioral engagement		T3 behavioral disaffection		T3 emotional engagement	
	*B* (SE)	*β*	*B* (SE)	*β*	*B* (SE)	*β*	*B* (SE)	*β*	*B* (SE)	*β*
Individual level										
Moderate trajectory	−0.04 (.08)	−.02	0.13 (.09)	.24	−0.01 (.08)	−.00	0.09 (.08)	.05	−0.00 (.08)	.00
High‐decreasing trajectory	−0.30 (.19)	−.08	0.19 (.08)*	.34	−0.11 (.11)	−.17	0.24 (.11)*	.31	−0.02 (.13)	−.02
Low‐increasing trajectory	−0.67 (.13)***	−.15	0.07 (.08)	.13	−0.15 (.08)^a^	−.23	0.26 (.11)*	.33	−0.22 (.08)**	−.33
Controls										
T1 outcomes	0.41 (.03)***	.42	0.44 (.05)***	.44	0.33 (.04)***	.35	0.29 (.04)***	.32	0.29 (.04)***	.34
Age	0.02 (.03)	.03	−0.03 (.02)	−.08	−0.02 (.03)	−.04	−0.08 (.04)*	−.12	0.00 (.02)	.00
School track: vocational	0.04 (.05)	.02	0.02 (.05)	.02	0.08 (.07)	.06	−0.03 (.11)	−.02	0.05 (.06)	.03
Gender	−0.07 (.07)	−.04	−0.14 (.05)**	−.12	−0.04 (.07)	−.06	−0.02 (.07)	−.03	−0.09 (.07)	−.14
Parental education: higher	0.07 (.10)	.03	0.00 (.06)	.00	0.02 (.07)	.01	−0.01 (.10)	−.00	0.06 (.06)	.03
*R* ^2^	.21		.24		.13		.13		.13	
School level										
Moderate school composition (30%–60%)	0.15 (.13)	.91	0.06 (.09)	.49	0.03 (.09)	.28	−0.13 (.08)	−1.23	0.10 (.11)	.78
High school composition (>60%)	0.16 (.14)	.99	0.02 (.09)	.13	−0.02 (.08)	−.21	−0.02 (.07)	−.23	0.06 (.10)	.44
*R* ^2^	.22		.05		.03		.32		.12	
Intraclass correlation	.03		.04		.02		.02		.03	

*Note*: The table presents unstandardized regression coefficients and their significance (with standard errors in parenthesis), along with standardized regression coefficients (STDYX). “Low‐discrimination” trajectory was the reference. *R* ^2^ at the school level indicates how much of the variance at the school level (as shown by the intraclass correlation) is explained by the model.

^a^ *p* = .072;

* *p* < .05;

** *p* < .01;

*** *p* < .001.

While we did not find any direct effects on school achievement (see Section S8), we examined a mediation model to explore if an indirect effect existed through engagement (Section S9). This revealed that adolescents experiencing increasing discrimination over 3 years became less engaged in school, subsequently leading to lower achievement. Finally, we replicated the analyses for school non‐compliance and behavioral disaffection, both of which had lower reliability, by defining those variables at the latent level to account for measurement error. Results replicated those reported here (Section S10).

## DISCUSSION

Using large‐scale multi‐level and longitudinal data (three waves, three cohorts), we examined change and stability in ethnically minoritized adolescents' experiences of ethnic discrimination in school. We took an ecological approach to adolescent adjustment to elucidate the evolving interethnic experiences in the school environment as a key developmental context (Bronfenbrenner & Morris, 2006; Eccles & Roeser, 2011). Given the dominance of U.S. samples in discrimination research, one of our study's key contributions lies in its exploration of an understudied research context and group. Our study is the first to establish individual trajectories of school‐based ethnic discrimination among ethnically minoritized adolescents in Europe. While most adolescents experienced low discrimination in school, we found that substantial proportions faced a more discriminatory school environment either initially or increasingly over time. Our contributions extend beyond this aspect. This is the first study to examine the influence of both individual and school‐level aggregated perceptions of school diversity climate on distinct trajectories of experienced discrimination. Through this, we demonstrated that minoritized adolescents attending schools with a stronger presence of ethnically minoritized peers and a more positive school diversity climate, emphasizing equality and cultural diversity, were less likely to follow less adaptive trajectories of discrimination such as those characterized by initially high or prolonged moderate levels of discriminatory experiences. Furthermore, our study is the first to investigate the association between these trajectories and multiple facets of school adjustment. Accordingly, ethnically minoritized adolescents encountering high discrimination at any point in their school careers, whether initially or subsequently, had worse school adjustment, as evidenced by lower school belonging (Niwa et al., 2014), reduced engagement, and higher non‐compliance. We discuss our contributions, limitations, and future implications next.

### Trajectories of ethnic discrimination

Our findings extend the limited evidence on trajectories of ethnic discrimination based on U.S. samples to discrimination experiences in the European school context (Lee et al., 2020; Niwa et al., 2014; Smith‐Bynum et al., 2014; Unger et al., 2016). In total, we identified four trajectories of experiences of discrimination in school. Most adolescents followed a low discrimination trajectory, replicating earlier findings with U.S. samples (Lee et al., 2020; Niwa et al., 2014; Smith‐Bynum et al., 2014; Unger et al., 2016). We also identified an initially high but decreasing discrimination trajectory, replicating another common trajectory found in earlier work (Lee et al., 2020; Niwa et al., 2014; Smith‐Bynum et al., 2014; Tynes et al., 2020; Unger et al., 2016). Finally, for some minoritized adolescents, their discrimination experiences worsened over time as evidenced by a low‐increasing trajectory of discrimination (Lee et al., 2020; Smith‐Bynum et al., 2014; Tynes et al., 2020; Unger et al., 2016). Despite the reliance in our study on repeated single‐item measures of discrimination, our findings thus replicate earlier studies that focused on general discrimination experiences and extended them to the school context. Earlier studies also found a negative trajectory of moderate or high‐stable discrimination in small subgroups (Lee et al., 2020; Smith‐Bynum et al., 2014; Unger et al., 2016). In our present study, this pattern was mostly resembled by the moderate trajectory that showed a small decrease over time, similar to Niwa et al. (2014). This could be related to the context of discrimination as school contexts are distinct from other contexts of discrimination. Schools might be more protective against discrimination, compared to, for instance, the internet where discrimination is very prevalent (Tynes et al., 2020).

Another unique factor could be the belief in school meritocracy. Contrary to the notion of an ideal and fair system grounded in individual merit, the prevalence of this belief poses a risk, potentially overshadowing principles of justice, need, and equality, and hindering rather than facilitating equal opportunity (Mijs, 2016). Consequently, this belief might complicate students' ability to recognize discrimination in schools. For instance, adolescents, who participated in a school‐based intervention that supports adolescents' ethnic‐racial identity in Germany, reported surprisingly more experiences of discrimination after the intervention (compared to the control group) (Juang et al., 2020). They reasoned that as the intervention involved discussions regarding discrimination, stereotyping, and racism, it might have also prompted more awareness among adolescents in the intervention group regarding unfair treatment in school. Moreover, our discrimination measure likely captured a more explicit recognition of unfair treatment as discrimination, given its direct phrasing. When coupled with school meritocracy beliefs, this might explain why we did not find a trajectory of high‐stable discrimination. Future research should replicate our findings using more fine‐grained measures and comparing experiences across different contexts of ethnic discrimination.

Sources of discrimination in school might also affect the discrimination trajectory that adolescents follow during their school years. Existing evidence, albeit limited, shows that discrimination by teachers or other school personnel is more strongly linked to lower school adjustment compared to discrimination by peers (Benner & Graham, 2013). While we could not model different trajectories for different sources of discrimination in school, we had information on whether adolescents experienced discrimination from their peers, teachers, or other personnel in school at time 1. We found that adolescents who reported more discrimination from teachers and other school personnel than from peers were more often in the high‐decreasing versus the moderate trajectory. While this provides some descriptive insight, more research is needed to clarify trajectories of discrimination by different sources, and their potentially different impact on adolescent development.

Embedding our findings within the Belgian school context, we have also looked at how our participants understood the grounds for their discrimination experiences in school. Religion, country, language, and skin color emerged as the top four cited grounds, in that order. This diversity of attributions reflects the nuanced discussions of race in Europe (Jugert et al., 2022; Moffitt et al., 2020) so that we can still interpret these attributions through ethnic or racial lenses. Even though our measure did not explicitly address ethnic or racial discrimination, it was the most common reason reported by adolescents. Future research should consider the intersectionality of these factors (Moffitt et al., 2020) and their link to distinct discrimination trajectories.

Our study also demonstrates how school structures can shape experiences of ethnic discrimination for ethnically minoritized students. In hierarchical tracking systems of Belgian secondary schools, students are streamed into different tracks to follow vocational, technical, or academic secondary education at an early age. Allocation into these tracks is based on earlier school performance, roughly separating students by their abilities. Importantly, minoritized students are more likely to be streamed into vocational education, even controlling for their early school success, compared to majority students (Baysu et al., 2018). When schools assign students to different tracks early on, it increases inequality because this amplifies earlier disadvantages in the home context. This occurs because students are separated into distinct tracks after few years of schooling, resulting in less time for those who begin with disadvantages in language and social skills—such as those from families with lower education or migration backgrounds—to bridge the gap (Baysu et al., 2018). These decisions subsequently determine students' future chances and are often irreversible (Baysu et al., 2018; Eccles & Roeser, 2011). Against this background, our findings show that ethnically minoritized students in vocational tracks are more likely to experience discrimination either initially or eventually (high‐decreasing and low‐increasing vs. the low‐discrimination trajectories). Given that, even in schools where ethnically minoritized students predominate, like vocational schools, ethnically minoritized teachers or other school personnel are still sparse (e.g., similar to the US context with minoritized African American adolescents, Chavous et al., 2008), one can speculate that this might add to minoritized students' sense of exclusion or discrimination. We need more research to understand the psychological mechanisms behind the higher exposure to discrimination experiences in vocational schools.

### School diversity climate and ethnic discrimination trajectories

While school environments as micro‐systems reflect interethnic relations in the wider society to some extent, school diversity climates vary considerably in how they represent, value, and manage ethnic diversity (Celeste et al., 2019). Besides replicating discrimination trajectories in the Belgian school context, another novel finding of our study is that perceived school diversity climates at time 1 mattered for the trajectories of school‐based ethnic discrimination over 3 years. When minoritized students perceived their school as more equal and multicultural, they were more likely to follow the low‐discrimination trajectory (vs. other trajectories). Since minoritized adolescents are at higher risk of experiencing discrimination in school (Bottiani et al., 2016), our finding that the perceptions of an inclusive school diversity climate may protect them from discrimination is encouraging. As reverse causation is less likely for longitudinal discrimination trajectories, our findings strengthen the recent evidence linking equality and multiculturalism to student experiences of discrimination (Benner & Graham, 2011) and school outcomes (Celeste et al., 2019; Schachner et al., 2019). We replicated some of these positive effects at the school level, so that shared student perceptions of equality made the low‐discrimination trajectory more likely (albeit marginally). Our findings are in line with findings of better‐quality relationships with teachers and better school outcomes in “fair” schools (Baysu et al., 2016, 2021; Schachner et al., 2019).

We also considered the relative proportion of ethnically minoritized and majority adolescents in school. Students in schools where ethnically minoritized students were the numerical majority (>60%) were more likely to be in the low‐discrimination trajectory (vs. moderate) than those in schools with fewer minoritized peers (<30%). This finding provides support to previous research suggesting a negative association between the share of ethnically minoritized pupils in school and experiences of discrimination (Agirdag et al., 2011; Juvonen et al., 2018), as opposed to research reporting a positive association (Benner & Graham, 2011; Brown & Chu, 2012). Thus, in the current study, a higher presence of minoritized peers protected adolescents against experiences of discrimination. We posit that a “majority‐minority” school environment may empower minoritized adolescents so that their relative numbers would protect them from becoming the targets of discrimination (Baysu et al., 2014).

Finally, looking at the specific discrimination trajectories, while perceived equality at both individual and school levels was protective against initially high (but decreasing) experiences of discrimination, perceived multiculturalism and higher presence of minoritized peers were protective against prolonged moderate levels of discrimination. While more research is needed to understand the effects of different aspects of school diversity climates on changing patterns of discrimination, taken together, the results highlight the significance of both objective (e.g., ethnic composition) and subjective (e.g., perceived equality and multiculturalism) aspects of the school ethnic context for minoritized students' experiences.

### Ethnic discrimination trajectories and later school adjustment

In light of the heightened risks of less favorable school careers among ethnically minoritized adolescents due to their ethnically minoritized status (Baysu et al., 2018), it is key to establish the developmental consequences of early or emergent ethnic discrimination experiences in school for changes in their school adjustment. Our findings thus contribute to the growing literature showing that discrimination predicts worse school outcomes among various minoritized groups. Experiencing low discrimination in school over 3 years (vs. high decreasing or low‐increasing trajectories) was associated with longitudinal changes in various school outcomes, in line with longitudinal evidence linking discrimination to poor school outcomes (Benner & Graham, 2011; Cheng et al., 2020; Hughes et al., 2016). Minoritized adolescents who were in the low‐discrimination trajectory reported significantly more school belonging, emotional and behavioral engagement, and school compliance, and less behavioral disaffection. While we did not find any direct effects of discrimination trajectories on achievement, further Supporting Information analyses revealed that adolescents experiencing low but increasing discrimination reported decreasing school engagement over time, subsequently leading to reduced achievement compared to those in the low‐discrimination trajectory. This implies that for individuals initially facing low discrimination, resorting to devaluation and disengagement could act as a coping mechanism against rising discrimination levels. However, this approach comes at a cost, manifested in lower achievement (Levy et al., 2016).

Of the five presented studies that focused on trajectories of ethnic discrimination (Lee et al., 2020; Niwa et al., 2014; Smith‐Bynum et al., 2014; Tynes et al., 2020; Unger et al., 2016), only Niwa et al. (2014) focused on a single school outcome, i.e., school belonging. They found that adolescents in the low‐stable group reported higher school belonging than those in the high‐decreasing discrimination. The findings of this study and the current study suggest that experiencing high levels of discrimination at any point during adolescence (either initially or eventually) might have detrimental consequences on school outcomes.

### Limitations and future directions

While our study addresses some of the gaps in the current research on ethnic discrimination experiences, we also acknowledge its limitations. First, our measure of school‐based discrimination consisted of only one item asking about the experience of discrimination, hostility, or unfairness in school. While our single‐item measure cannot fully capture ethnic discrimination as a complex social phenomenon that occurs in various ways during interactions in different contexts, our results replicated trajectories in other studies using multiple items, including “being called names” (Niwa et al., 2014), “being ignored or not taken seriously,” or “being treated rudely or disrespectfully” (Lee et al., 2020; Smith‐Bynum et al., 2014; Unger et al., 2016). Moreover, our measure does not explicitly refer to ethnic‐based discrimination, even though it was the most common reason adolescents reported in our follow‐up questions. Future studies should use multiple items distinguishing more subtle versus explicit forms of discrimination, different discrimination contexts (school vs. other contexts, e.g., Niwa et al., 2014), grounds (Moffitt et al., 2020), and sources of discrimination (e.g., Benner & Graham, 2013) to further disentangle the social complexity of these experiences. Second, although the current study examined longitudinal associations, our findings do not indicate causality or rule out the possibility that these associations could be bi‐directional. Thus, discrimination experiences might be seen as reactive in response to worsening school outcomes, although studies testing the directionality find more support for the path from discrimination to school outcomes rather than the other way around (Cheng et al., 2020). Relatedly, while we theorize that the school climate shapes individual experiences of discrimination, discrimination experiences might also shape students' perceptions of the school diversity climate. Future studies should use more external measures of school climate such as actual school‐level policies and practices favoring different diversity approaches (Celeste et al., 2019). Third, the short lengths of certain measures, such as student‐reported non‐compliance, behavioral disaffection, and equality, made it more problematic to evaluate reliability with Cronbach's alpha (Sijtsma, 2009). CFAs and additional analysis with compliance and behavioral disaffection as latent outcomes suggested that the items describe a common construct and confirmed the results reported here. Moreover, positive associations between ethnically minoritized students' perceptions of equality and discrimination trajectories were replicated at the school level. This approach which measured shared (rather than individual) student perceptions had better reliability, strengthening the conclusions regarding this measure. Fourth, our attrition analysis revealed that participants who missed at least one data collection wave reported increased discrimination and poorer school adjustment at time 1. Although this finding still aligns with the MAR assumption, for which we applied FIML as a robust and unbiased approach, future studies should compare alternative models for dealing with missingness in longitudinal studies (see Section S5 for further discussion).

Fifth, while we used parentage as a frequently‐used indicator of immigration background in Belgium and in Europe at large, we also recognize the importance of ethnic‐racial self‐identification (Jugert et al., 2022). Since minoritized youth's self‐identification correlates with higher perceived ethnic discrimination (e.g., Fleischmann et al., 2011 among minoritized youth of Turkish and Moroccan background in Europe), using self‐identification as an inclusion criterion might have limited the range of captured discrimination experiences, potentially excluding those with lower or no discrimination perceptions. Nonetheless, future studies should use diverse ethnic‐racial group definitions to explore links between youth's self‐identification versus parentage and their ethnic discrimination experiences. Relatedly, our decision to focus on these two ethnically minoritized groups was because of their similarities—such as arrival period, legal conditions, and cultural and religious attributes—coupled with their status as the most disadvantaged and stigmatized groups in Belgium. This focused approach enabled us to overcome challenges that result from aggregating highly diverse minoritized groups into a single category. While our study did not yield significant effects of Turkish or Moroccan background on discrimination trajectories or school outcomes, research suggests that adolescents from European‐immigration backgrounds, who experience lower levels of stigmatization in Belgium, may exhibit distinct school experiences (Hillekens et al., 2023). Thus, future studies should emphasize comparisons across different groups who face varying degrees of stigmatization (Hillekens et al., 2023; Schwarzenthal et al., 2024).

## CONCLUSION

Growing empirical research documents the negative consequences of ethnic discrimination on academic outcomes during adolescence. However, this literature is heavily weighted to US contexts and samples and often focuses on experienced discrimination at one point in time or in retrospect or its average change across minoritized adolescents over time. Addressing these research gaps, our study elucidated different trajectories of change and stability in school‐based ethnic discrimination among ethnically minoritized adolescents in Europe. Moreover, the current study showed that even transient experiences of high discrimination at any one point in the school career (initially or eventually) were associated with worse school outcomes later. By implication, any discriminatory episodes can cast a long shadow over future chances for ethnically minoritized students to succeed.

At the same time, perceptions of a positive school diversity climate at the individual and school levels along with a high presence of ethnically minoritized peers protected minoritized adolescents from experiencing persistent or initially high discrimination in school over time. Our findings thus highlight the long‐term benefits of promoting fairness and diversity in schools as key buffers against discrimination experiences, and in turn, as a way to help ethnically minoritized students succeed in school. Promoting diversity and combating discrimination in educational institutions lays the ground for broader social change through fostering tolerance, reducing prejudice, and challenging stereotypes in the larger community.

From an applied perspective, these findings suggest that schools can implement evidence‐informed diversity interventions to protect at‐risk minoritized adolescents from adverse outcomes. Such interventions should enhance the ethnic diversity climate in school through engaging not only students but also teachers and staff who are jointly shaping the school diversity climate. Comprehensive interventions may cover a range of diversity‐related challenges and competencies, including fostering an understanding and appreciation of diversity and differences, as well as the ability to recognize and address discriminatory behaviors, stereotypes, and prejudice.

## FUNDING INFORMATION

This research is supported by grants from the Research Foundation‐Flanders (Fonds Wetenschappelijk Onderzoek‐FWO G.0747.13), University of Leuven Research Council (Interdisciplinaire Onderzoeksprogramma‐IDO 10.005), Jacobs Foundation and the Advanced Research Collaborative (ARC) Distinguished Visiting Fellowship the Graduate Center CUNY.

## CONFLICT OF INTEREST STATEMENT

We have no conflict of interest to disclose.

## Supporting information


Appendix S1.

## Data Availability

The data and code necessary to reproduce the analyses presented here are publicly accessible, as are the materials necessary to attempt to replicate the findings. Analyses were not pre‐registered. Data, code, materials, and the preregistration for this research are available at the following URL: link.
