# Supplementary material for: Trajectories of ethnic discrimination and school adjustment of ethnically minoritized adolescents: The role of school diversity climate
Source: Child Dev. 2024 Aug 11;95(6):2215–31. doi: 10.1111/cdev.14133 (PMC11579631; doi:10.1111/cdev.14133)
Supplement: Supplementary file 1 — Appendix S1. [file CDEV-95-2215-s001.docx]

**Supplementary Online Materials**

Additional descriptive analyses, model specifications, and further technical information about models and analyses, replications, and alternative models were presented in this section.

Contents

[Section 1. Reasons for and Sources of Discrimination 2](#_Toc168394639)

[Section 2. Additional Information on the Psychometric Properties of the Scales 4](#_Toc168394640)

[Measurement Invariance Testing 6](#_Toc168394641)

[Section 3. Additional Information on Model Specifications and Fit 7](#_Toc168394642)

[Predicting Ethnic Discrimination Trajectories from School Diversity Climate 8](#_Toc168394643)

[Predicting Academic Outcomes from Ethnic Discrimination Trajectories 8](#_Toc168394644)

[School belonging model 8](#_Toc168394645)

[School non-compliance model 9](#_Toc168394646)

[School engagement models 10](#_Toc168394647)

[Section 4. Detailed Comparison with Past Publications 11](#_Toc168394648)

[Section 5. Missingness Assumptions 13](#_Toc168394649)

[Section 6. Latent Growth Curve Models: Overall Trend in Ethnic Discrimination 13](#_Toc168394650)

[Section 7. Model Fit Statistics for Latent Growth Mixture Models 14](#_Toc168394651)

[Section 8. The Effects of Ethnic Discrimination Trajectories on School Achievement 16](#_Toc168394652)

[Section 9. The Indirect Effects of Ethnic Discrimination Trajectories on School Achievement via Engagement 17](#_Toc168394653)

[Section 10. Latent Outcome Models 20](#_Toc168394654)

[Latent non-compliance model 20](#_Toc168394655)

[Latent behavioral disaffection 20](#_Toc168394656)

[Section 11. Positionality Statement 23](#_Toc168394657)

[References (not cited in the manuscript) 23](#_Toc168394658)

# Section 1. Reasons for and Sources of Discrimination

Most adolescents in our sample (n = 1002, 69.3%) said they were not being discriminated against at time 1. The remaining part reported that they were being discriminated against sometimes (n = 228, 15.8%), often (n =53, 3.7%), or almost always (n = 34, 2.4%) (besides, n = 128, 8.9% left the question unanswered, thus missing). Those who reported that they experienced at least some discrimination (n = 315, 22% of all participants, 24% of the valid responses) were asked about the reasons and sources of discrimination. Percentages presented in the paper under Measures are not cumulative as participants were allowed to choose more than one reason or source. We then created a dummy-coded variable to indicate ethnically-motivated discrimination (=1 if respondents mentioned any of the most-evidently ethnic reasons, i.e., country, religion, language, skin color). We also re-coded the sources of discrimination as a three-category variable: only by their teachers/school personnel, only by peers, and by both. While Table S1 below reports percentages of all participants, focusing only on the valid responses from those 315 participants who experienced at least some discrimination, 65% indicated ethnic reasons, 35% indicated their peers as sources or perpetrators of discrimination, 43% indicated their teachers/school personnel, 22% indicated both

Table S1. Descriptives for reasons and sources of discrimination

| Reasons for Discrimination | | | |
| --- | --- | --- | --- |
|  | Frequency | Percent | Valid Percent |
| Not discriminated | 1002 | 69.3 | 76.5 |
| Discriminated non-ethnic reasons | 109 | 7.5 | 8.3 |
| Discriminated ethnic reasons | 198 | 13.7 | 15.1 |
| Total | 1309 | 90.6 | 100 |
| Missing | 136 | 9.4 |  |
| total | 1445 | 100 |  |
| Sources for Discrimination | | | |
|  | Frequency | Percent | Valid Percent |
| Not discriminated | 1002 | 69.3 | 80.2 |
| Peers | 88 | 6.1 | 7 |
| School and teachers | 106 | 7.3 | 8.5 |
| Both | 54 | 3.7 | 4.3 |
| Total | 1250 | 86.5 | 100 |
| Missing | 195 | 13.5 |  |
| Total | 1445 | 100 |  |

Next, we looked at how these reasons (Table S2.a) and sources of discrimination (Table S2.b) were represented in each discrimination trajectory. Overall, in both the moderate and the high-decreasing trajectories, most students have been discriminated against for ethnic reasons (Table S2.a). As for the sources of discrimination, in the moderate group, discrimination was equally perpetrated by either peers or school personnel, however, in the high-decreasing group teacher/school personnel were the main perpetrators of discrimination at time 1. Whilst this provides some descriptive insight into the issue, we could not analyze these differences statistically due to small sample sizes.

**Table S2.a** *Reasons for discrimination in each discrimination trajectory*

| *Discrimination trajectories* | | **Moderate** | **High-decreasing** | **Low-increasing** | **Low** |
| --- | --- | --- | --- | --- | --- |
| *Discrimination perpetrator*  *(at T1)* | Not discriminated | 0.0% | 0.0% | 96.6% | 99.1% |
|  | Other reasons | 34.9% | 36.7% | 0.0% | 0.5% |
|  | Distinctly ethnic reasons | 65.1% | 63.3% | 3.4% | 0.4% |
|  | *n* | 212 | 79 | 58 | 955 |

**Table S2.b** *Perpetrators of discrimination in each discrimination trajectory*

| *Discrimination trajectories* | | **Moderate** | **High-decreasing** | **Low-increasing** | **Low** |
| --- | --- | --- | --- | --- | --- |
| *Discrimination perpetrator*  *(at T1)* | Not discriminated | 0.0% | 0.0% | 98.2% | 99.4% |
|  | Peers | 40.8% | 21.5% | 0.0% | 0.3% |
|  | Teacher/school personnel | 40.8% | 47.7% | 1.8% | 0.2% |
|  | Both | 18.4% | 30.8% | 0.0% | 0.1% |
|  | *n* | 174 | 65 | 57 | 952 |

# Section 2. Additional Information on the Psychometric Properties of the Scales

We performed a confirmatory factor analysis (CFA) using MLR estimator to establish the psychometric properties of all study measures consisting of at least two items, i.e., perceived equality (two items) and perceived multiculturalism in school (four items), school belonging (four items), behavioral engagement (three items), emotional engagement (three items), disengagement (three items), and school non-compliance (three items). The CFA showed a good fit, RMSEA = .04, CFI = .93. We found that the standardized loadings ranged from 0.43 to 0.87, i.e., all were higher than the cut-off 0.40 suggested by Stevens (1992), and 15 out of the 22-factor loadings were above 0.60, i.e., could be assessed as very good or excellent (Comrey & Lee, 1992), and all were also significant at p < .001 (Table S3). As seen in Table S4, the individual factors representing the measures had generally weak to moderately strong relationships (standardized covariance below 0.60), with the exception of school belonging with emotional engagement (std. cov. 0.68) and behavioral engagement with disengagement (std. cov. 0.61). This was not surprising given the similarities between these concepts on a theoretical level, however none of the standardized covariances was strong enough to suggest that the factors would measure the same concept. Similar results were obtained when we conducted the CFA with Time 3 items (without perceived multiculturalism, which we only measured at T1). We present the standardized factor loadings and covariances below.

**Table S3**. *Standardized CFA factor loadings for study measures at T1*

|  | Loadings | SE |
| --- | --- | --- |
| **T1 School belonging** |  |  |
| I am proud to be a student of this school | 0.77 | 0.02 |
| I feel happy at this school | 0.86 | 0.01 |
| I feel at home at this school | 0.75 | 0.02 |
| I would prefer to go to another school (reverse item) | 0.68 | 0.02 |
| **T1 Behavioral engagement** |  |  |
| I work as hard as I can in class | 0.68 | 0.02 |
| I listen carefully during the class | 0.82 | 0.02 |
| I pay attention in class | 0.82 | 0.02 |
| **T1 Emotional engagement** |  |  |
| I like to learn new things in class | 0.54 | 0.03 |
| I feel good in class | 0.76 | 0.02 |
| I like to be in class | 0.80 | 0.02 |
| **T1 Behavioral disengagement** |  |  |
| I often think of other things during class | 0.61 | 0.04 |
| I do not really do my best at school. | 0.55 | 0.04 |
| In class I am easily distracted (in class my thoughts easily wander away) | 0.65 | 0.04 |
| **T1 School non-compliance** |  |  |
| This school year, how often did it occur that ….  … you got a punishment in school (e.g. being sent out of class, detention-work)? | 0.73 | 0.03 |
| …you skipped class? | 0.43 | 0.04 |
| …you were late to school? | 0.52 | 0.04 |

*Note*. All loadings significant at p < .001.

**Table** S4. *Standardized factor covariances*

|  | 1 | 2 | 3 | 4 | 5 | 6 |
| --- | --- | --- | --- | --- | --- | --- |
| T1 Perceived equality in school |  |  |  |  |  |  |
| T1 Perceived multiculturalism | 0.55^***^ |  |  |  |  |  |
| T1 School belonging | 0.56^***^ | 0.34^***^ |  |  |  |  |
| T1 Behavioral engagement | 0.30^***^ | 0.20^***^ | 0.33^***^ |  |  |  |
| T1 Emotional engagement | 0.46^***^ | 0.30^***^ | 0.68^***^ | 0.53^***^ |  |  |
| T1 Behavioral disengagement | -0.39^***^ | -0.11^*^ | -0.31^***^ | -0.61^***^ | -0.35^***^ |  |
| T1 School non-compliance | 0.36^***^ | 0.15^***^ | 0.35^***^ | 0.51^***^ | 0.37^***^ | 0.59^***^ |

*Note. * p* < .05, ** *p* < .01, *** *p* < .001

### Measurement Invariance Testing

We performed a multigroup analysis where we assessed the measurement invariance in the Turkish versus the Moroccan-background groups. We used the robust maximum likelihood estimator (MLR), and we compared the nested models fit via scaled chi2 difference testing as recommended on the Mplus website (Muthen & Muthen, 2023). We initially estimated a configural model, in which we fixed factor means to 0 and all factor variances to 1 for identification. We then assessed whether the fit of the model worsened when all factor loadings were set as equal in both groups (metric invariance) (Table S5). Based on the difference testing, we found that the metric invariance model did not have significantly worse fit, chi2(22) = 18.09, *p* = .701. In the next step, we also restricted all intercepts to hold them equal across both groups (scalar invariance). The scalar invariance model had slightly worse fit compared to the metric invariance model, and this difference was statistically significant, chi2(22) = 56.38, *p* < .001. However, the modification indices suggested that allowing three of the intercepts to vary across groups might improve the fit. We therefore freed the intercept of three items: one from the school belonging scale (“I feel happy at this school”) and two from the Behavioral disengagement scale (“I often think of other things during class “, “I do not really do my best at school.”). This significantly improved the model fit compared to the full scalar model, chi2(3) = 33.65, *p* < .001, and this model did not have significantly different fit compared to the metric model, chi2(19) = 23.43, *p* = 219. Nevertheless, the differences in item intercepts were only small, for “I feel happy at this school”, the intercept was 3.61 in the Moroccan-background group vs. 3.70 in Turkish-background group; for “I often think of other things during classes”, the intercept in the Moroccan-background group was 3.11 vs. 3.00 in the Turkish-background group; and for “I do not really do my best at school.”, the intercept in the Moroccan-background group was 1.98 vs. 2.21 in the Turkish-background group. Overall, we found that psychometric qualities of the scales were comparable in both groups. All models had a good fit, with RMSEA between .04 - .05, and CFI = .92 (Table S5).

**Table S5.** *Comparison of model fit for invariance testing*

| **Model** | **Chi2** | **Chi2 scale factor** | **Chi2 DF** | **RMSEA** | **CFI** |
| --- | --- | --- | --- | --- | --- |
| Configural | 947.20 | 1.16 | 376 | 0.05 | 0.92 |
| Metric invariance | 959.13 | 1.17 | 398 | 0.04 | 0.92 |
| Scalar invariance | 1015.06 | 1.16 | 420 | 0.04 | 0.92 |
| Partial Scalar | 985.55 | 1.16 | 417 | 0.04 | 0.92 |

# Section 3. Additional Information on Model Specifications and Fit

Mplus does not automatically include variances and means of the predictors to estimate the missing data on predictor (X) variables. To account for missing data, one can use full information maximum likelihood (FIML) and model the variances and means of the predictors. Mplus does not automatically include the covariances between predictors either. We added correlated covariances based on theoretical expectations when there is such an expectation (for instance, between fairness and multiculturalism in the predictor models). When there was no a priori theoretical expectation such as between control variables, we added correlated covariances based on modification indices, which is a widely used strategy to improve model fit, as long as these covariances were also theoretically sound. Note that model fit suffers when covariances between significantly correlated predictors are excluded. We only kept significant covariances for having a simpler model (i.e., fewer parameters to estimate). Given that the models are very complex and have a high number of parameters, our choice to include only the significantly associated covariances rather than the including all possible covariances opts for more parsimonious/simpler models and allows us to run multilevel models (where one is constrained by the rule where the number of parameters cannot exceed the number of clusters). Thus, to improve model fit, we modeled covariances between predictor variables by looking at modification indices until CFI was considered acceptable (CFI > .80). Overall, by including the covariances, we were able to improve the model fit, and the results remained comparable to the simpler model (without modeled covariances).

## Predicting Ethnic Discrimination Trajectories from School Diversity Climate

To account for missing data, we modeled the following variances at the individual level: age, vocational track, gender, parental education, perceived equality, and multiculturalism in school at time 1. We also modelled the covariance between perceived equality (E) and multiculturalism (M) at time 1, standardized cov(E, M) = 0.28, p < .001. Mplus does not provide the usual model fit statistics (RMSEA and CFI) for categorical outcomes.

## Predicting Academic Outcomes from Ethnic Discrimination Trajectories

In all school outcome models, we modeled the variances of age, school track, parental education, and time 1 (T1) outcomes at the individual level to account for missing data. All school outcome models had a satisfactory model fit with RMSEA <= 0.05, CFI >= 0.84. While the CFI is lower in some outcome models than the traditional 0.90 cutoff, the RMSEA showed acceptable to good model fit throughout. Slightly lower CFI is likely due to the complexity of the models for which CFI punishes more severely (Shi et al., 2019).

We provide a detailed specification for each model below:

### School belonging model

We estimated the variances for the following T1 variables: age, gender, moderate and high-decreasing discrimination trajectories, school belonging, and parental education. We also included the variances of the vocational track and the low-increasing discrimination trajectory, however, due to model non-identification, these could not be freely estimated. Instead, we restricted the values of these (modeled) variances to match the sample variances.

We also modeled the following covariances between T1 school belonging (SB) and: (1) age cov(SB, age) = -0.20, (2) Moderate trajectory, cov(SB, moderate) = -0.05 , (3) High-decreasing trajectory, cov(SB, high) = -0.02 , (4) Gender, cov(SB, gender) = 0.05 , all *p*s < .01. According to these covariances, older adolescents, those who followed either the moderate or the high-decreasing discrimination trajectory and boys had significantly lower school belonging at time 1. The covariances between parental education (PE) and (1) Gender, cov(PE, girl) = -0.02, and (2) Age, cov(PE, age) = -0.04, both *p*s < .01, were also modeled, suggesting that the parents of boys and younger adolescents had slightly higher qualifications. We also modeled the covariances between the moderate-discrimination trajectory and both the (1) high-decreasing cov(moderate, high) = -0.01, and the (2)low-increasing discrimination trajectories, cov (moderate, increasing) = -0.07, both *p*s < .001. The model had a good fit, RMSEA = 0.02 and CFI = 0.96.

### School non-compliance model

We estimated the variances for the following T1 variables: age and gender. We also included the variances of the vocational track, parental education, and T1 school noncompliance, however, due to model non-identification, these could not be freely estimated. Instead, we restricted the values of these (modeled) variances to match the sample variances.

We additionally modelled the following covariances between T1 school noncompliance (NC) and: (1) age cov(NC, age) = 0.10, (2) vocational track, cov(NC, vocational)= 0.04 , (3) gender, cov(NC, girl) = -0.03 , all *p*s < .001. The covariances suggest that older adolescents, those attending vocational education, and boys had more school problems at T1. The fit of the school non-compliance model was acceptable, RMSEA = 0.03 and CFI = 0.84.

### School engagement models

We modeled the variances of the following T1 variables: age, parental education, and the three dimensions of T1 engagement. The variances of the vocational track and the moderate discrimination trajectory were also brought into the model, both variances were restricted to sample variances to avoid error in parameter estimation.

The engagement model focused on the three dimensions of engagement, each representing a distinctive outcome. We included the covariances between the individual dimensions of engagement (Table S6) since the dimensions of engagement all theoretically represent a common underlying concept. While we modeled the covariances between the T3 engagement dimensions both at the individual (within) and school (between) level, none of the between level covariances reached statistical significance. We also modeled the covariances between T1 emotional engagement (EE) and (1) moderate trajectory, cov(EE, moderate)= -0.03, *p* < .001 and (2) Vocational track, cov(EE, vocational) = 0.03, *p* = .014. The covariances suggest that students in the low discrimination trajectory (vs. the moderate trajectory) and those studying in vocational schools had a slightly higher emotional engagement in school at T1. This model had an acceptable fit, RMSEA = 0.05, CFI = 0.86.

**Table S6.** *Covariances between the three dimensions of school engagement at time 1 and time 3*

|  | Time 1 | |  | Time 3 | |
| --- | --- | --- | --- | --- | --- |
|  | BE | BD |  | BE | BD |
| Behavioral engagement |  |  |  |  |  |
|  |  |  |  |  |  |
| Behavioral disaffection | -0.26^***^ |  |  | -0.13^***^ |  |
|  |  |  |  | -0.01 |  |
| Emotional engagement | 0.24^***^ | -0.18^***^ |  | 0.16^***^ | -0.04^a^ |
|  |  |  |  | 0.01 | -0.01 |

** p* < .05, ** *p* < .01, *** *p* < .001, ^a^ *p* = .052. *Note.* T3 covariances are residual covariances, as those are dependent variables. T3 covariances at the top and bottom rows refer to within and between level covariances respectively.

# Section 4. Detailed Comparison with Past Publications

Most relevant to the current study are the following two publications (Baysu et al., 2021; Hillekens et al, 2023). While Hillekens et al. (2023) focus on trajectories of acculturation, our current study focuses on trajectories of ethnic discrimination; thus our current study uses new data (repeated ethnic discrimination measures, new school diversity climate measures, and some new dependent measures) and has a different focus (on minoritized Turkish- & Moroccan-background samples & experiences). While Baysu et al (2021) focus on trajectories of teacher support and rejection, our current study focuses on trajectories of ethnic discrimination and thus uses new data (repeated ethnic discrimination measures, some new climate measures) and has a different focus (on minoritized Turkish & Moroccan-background samples & experiences). Table S7 provides a more detailed comparison between the current study and these previous publications.

**Table S7**. Data transparency table comparing constructs and methods between this manuscript and the past relevant papers

|  | Hillekens et al 2023 | Baysu et al 2021 | This manuscript |
| --- | --- | --- | --- |
| Main focus |  |  |  |
| Ethnic discrimination |  |  | x |
| Reasons for discrimination |  |  | x |
| Sources of discrimination |  |  | x |
| Cultural heritage maintenance | x |  |  |
| Cultural adoption | x |  |  |
| Teacher support |  | x |  |
| Teacher rejection |  | x |  |
| School outcomes |  |  |  |
| School belonging | x | x | x |
| School engagement | x | x | x |
| Achievement | x | x | x |
| School compliance |  | x | x |
| Contextual antecedents |  |  |  |
| Student perceptions of perceived equality |  | x | x |
| Student perceptions of multiculturalism |  | x | x |
| Ethnic composition | as a control |  | x |
| Teacher perceptions of multiculturalism |  | x |  |
| Teacher perceptions of assimilationism |  | x |  |
| Analysis |  |  |  |
|  | Trajectories of acculturation (LGMM) | Trajectories of teacher support and rejection (LGMM) | Trajectories of discrimination  (LGMM) |
| Sample |  |  |  |
|  | Minoritized adolescents (European, Turkish, and Moroccan-background) | Majority and minoritized adolescents (Turkish and Moroccan-background) | Minoritized adolescents (Turkish and Moroccan-background) |

# Section 5. Missingness Assumptions

We used FIML to account for missingness. Our attrition analysis showed that those who missed at least one wave of data collection reported more discrimination and lower school adjustment at time 1. This finding is in line with the MAR (Missing at Random) assumption where missingness can depend on either the previous history of the variables or on the other observed variables, for which the FIML method we used was found to be robust and unbiased. However, as there is no test for MNAR (missing not at random), there is a chance that for example, missingness could be a function of growth factors (MNAR), a risk associated with conducting longitudinal growth models. There are some methods that could be used under the MNAR assumption (e.g., using a pattern mixture model or the Diggle-Kenward selection model). For example, Muthen et al. (2011) conducted such a comparison, concluding that the results of mixture models under MAR and MNAR yielded similar trajectories. While comparing the findings from the MAR vs. MNAR models would be beyond the scope of this paper, future studies on ethnic discrimination trajectories should take into account alternative approaches to missingness.

# Section 6. Latent Growth Curve Models: Overall Trend in Ethnic Discrimination

We examined the overall trend in ethnic discrimination by estimating the average intercept and slope in a multilevel latent growth model. Students had generally low, slightly increasing levels of discrimination, *I* = 1.35, *p* < .001, *S* = 0.06, *p* = .009. Moreover, there was significant variability among students’ initial experiences of discrimination at time 1, and in the change in discrimination over time - as suggested by significant variances in slopes and intercepts (both *ps* = .004). At the school level, there were significant differences between schools’ initial levels of discrimination (*p* = .022) but not in how it changed over time (*p* = .665).

# Section 7. Model Fit Statistics for Latent Growth Mixture Models

Visual representation of the fit statistics (Figures S1-S2) shows that the 3-class solution fits the data better than the 2-class solution and that the model fit continued improving slightly beyond the 3-class solution. While the fit statistics show a clear breaking point at the 3-class solution, the fourth trajectory (a low-increasing trajectory) provided important insight and was theoretically justified; also, the 4-class solution was still a better fitting model statistically. Figures S3-S4 show that a low-increasing trajectory is missing in a 3-class solution, but it shows up in a 4-class solution (presented in the paper) as well as in a 5-class solution (see below). The 5-class solution divided the moderate group further into two groups, one of them consisting of only 1.4% of adolescents. We, therefore, chose a 4-class solution as our final model.

The models (1- to 5-class solution) have been specified as mixture models with a fixed number of random starts/LRT-starts. In the 5-class solution, we used the OPTSEED option with no random starts to save computational time and avoid error messages. This “uses the starting values that gave the best loglikelihood so that no random perturbation of the starting values is needed” (Asparouhov & Muthen, 2012, p.9).

**Figure S1-S2** *Fit statistics for ethnic discrimination trajectories*

**Figures S3-S4.** *3 and 5 class trajectory solutions*

# Section 8. The Effects of Ethnic Discrimination Trajectories on School Achievement

We also investigated a model with school achievement outcomes (Math and Dutch scores). This model had a good fit, RMSEA = 0.03 and CFI = 0.96. It included the variances and means for age, vocational track, parental education, Math, and Dutch grades at time 1 as well as the positive covariance between Math and Dutch grades at time 1. None of the main study variables had significant effects on grades (see Table S8).

**Table S8** *Predicting school achievement at T3 by* *ethnic discrimination trajectories* *and age*

|  | *Outcome:* | **T3 Maths score** | | | | | | | **T3 Dutch score** | | | | |
| --- | --- | --- | --- | --- | --- | --- | --- | --- | --- | --- | --- | --- | --- |
|  | *Predictors:* | ***B*** | | ***SE*** | | | ***β*** |  | ***B*** | | ***SE*** | | ***β*** |
|  | **Within level** |  |  | |  |  | | |  |  | |  |  |
| *Main effects* | Moderate | -1.79 | | 1.63 | | | -0.05 |  | -2.57 | | 1.67 | | -0.08 |
|  | High-decreasing | -2.80 | | 1.96 | | | -0.05 |  | 2.30 | | 2.16 | | 0.05 |
|  | Low-increasing | -0.64 | | 2.27 | | | -0.01 |  | 1.56 | | 1.47 | | 0.03 |
| *Controls* | T1 outcome | 0.14^*^ | | 0.06 | | | 0.26 |  | 0.11^*^ | | 0.05 | | 0.22 |
|  | Age | 0.17 | | 0.59 | | | 0.02 |  | -0.28 | | 0.56 | | -0.03 |
|  | Gender | -1.67 | | 1.27 | | | -0.06 |  | 1.69 | | 1.26 | | 0.07 |
|  | Parental education | 2.16 | | 1.69 | | | 0.06 |  | 1.48 | | 1.37 | | 0.05 |
|  | *R^2^* | 0.13 | |  | | |  |  | 0.11 | |  | |  |
|  | **Between level** |  | |  | | |  |  |  | |  | |  |
|  | Moderate school composition (*30-60%)* | -1.53 | | 2.08 | | | -0.29 |  | -0.88 | | 1.62 | | -0.17 |
|  | High school composition (*> 60%)* | -1.14 | | 1.83 | | | -0.18 |  | -1.92 | | 1.68 | | -0.31 |
|  | *R^2^* | 0.08 | |  | | |  |  | 0.09 | |  | |  |
|  | Intraclass correlations | 0.09 | |  | | |  |  | 0.06 | |  | |  |

** p* < .05, ** *p* < .01, *** *p* < .001.

# Section 9. The Indirect Effects of Ethnic Discrimination Trajectories on School Achievement via Engagement

We explored whether ethnic discrimination trajectories had indirect effects on school achievement via reduced engagement. It is reasonable to expect such a mediation: School disengagement, or the reduction or absence of effort (Buhs et al., 2006), predicts lower achievement (Fredricks et al., 2004). There is also preliminary evidence suggesting (dis)engagement from learning or from the task as a key process connecting minoritized adolescents’ experiences of discrimination or exclusion in school to underachievement (Baysu et al., 2016; Buhs et al., 2006). Thus, we tentatively expected that minoritized adolescents in low-discrimination trajectories might be less at risk of disengaging from school, which may, in turn, protect their school achievement over time. We also explored which high discrimination trajectory would be more costly in terms of school engagement and in turn achievement.

We tested whether school engagement (T3) mediated the association between the ethnic discrimination trajectories and school achievement (T3), controlling for T1 engagement and achievement. In this model, instead of defining the three dimensions of engagement as separate variables, we modeled engagement as one latent variable, representing students’ overall engagement in school. We did this for both the T1 and the T3 engagement. Similarly, school achievement at T1-T3 were also defined as latent variables (based on Math and Dutch scores), representing students’ overall achievement in school. This model, therefore, included school engagement as a (latent) mediator between discrimination trajectories and T3 school achievement (latent).

In terms of model specifics, the direct effects of discrimination trajectories on achievement were first included in the model but were then removed as they were not significant (all ps >.10) in line with the non-significant results in the model predicting grades directly. We modeled the variances of the following T1 observed variables: age and parental education, the variance of both latent factors (T1 engagement and T1 achievement), and their covariance was included as a default. The covariance, cov(T1eng., T1achiev.)= 2.02, *p* = .006, suggests that higher engagement at T1 was associated with higher T1 achievement. We also modeled the residual covariance between T1 behavioral engagement and disaffection under the (theoretical) assumption that these two dimensions of engagement both represent the behavioral aspect (rather than emotional) and therefore there might be some covariance between them not explained by the engagement factor, however, this covariance was not statistically significant, *p* = .929. At the between level, we only identified T3 engagement and achievement as latent variables and modeled their covariance (p = .522).

The latent model had an acceptable fit, RMSEA = 0.05, CFI = 0.85. As can be seen in Table S9, all factor loadings in the latent model were strong (> 0.5) and statistically significant. The indirect effect was calculated at the within-level as a (the effect of the predictor on the mediator) multiplied by b (the effect of the mediator on the outcome variable), i.e. ind = a*b (Preacher et al., 2010, 2011).

As seen in Table S10, we found that minoritized adolescents in the low-discrimination trajectory had significantly higher school engagement than those in the low-increasing group at T3 (*B* = -0.21, *SE* = 0.08, *p* = .005, β = -0.07). In turn, more school engagement at T3 predicted significantly higher achievement at T3 (*B* = 3.92, *SE* = 1.20, *p* = .002, β = 0.25). The (individual-level) indirect effect of the low-increasing trajectory on achievement via reduced engagement was significant, *t* = -2.81, *p* =.005. No other trajectory had significant effects on engagement or achievement. The model explained 38% of the variance in engagement and 14% in achievement at the individual level. In summary, adolescents who experienced a gradual increase in discrimination experiences over three years became less engaged in school, and in turn, had lower achievement

**Table S9.** *School engagement and achievement at time 3 as latent outcomes predicted by ethnic discrimination trajectories: Standardized factor loadings (with standard errors in parentheses) and intercepts*

|  | T1 School engagement | |  | T3School engagement | | | |
| --- | --- | --- | --- | --- | --- | --- | --- |
|  |  | |  | *Within-level* | *Between level* | |  |
|  |  | Intercept |  |  |  | Intercept |  |
|  |  |  |  |  |  |  |  |
| T1 Behavioral engagement | 0.82 (0.05) | 5.50 |  | 0.86 (0.04) | 0.96 (0.05) | 101.73 |  |
| T1 Behavioral disaffection | -0.56 (0.06) | 3.28 |  | -0.50 (0.06) | 0.98 (0.06) | 63.62 |  |
| T1 Emotional engagement | 0.54 (0.04) | 4.81 |  | 0.51 (0.06) | 0.99 (0.03) | 59.66 |  |
|  | T1 School achievement | |  | T3 School achievement | | | |
| T1 Math | 0.89 (0.03) | 2.41 |  | 0.70 (0.06) | 1.00 (0.00) | 28.54 |  |
| T1 Dutch | 0.92 (0.03) | 2.34 |  | 0.78 (0.04) | 1.00 (0.00) | 28.18 |  |

*Note*. All factor loadings and significant at *p* < .001.

**Table S10** *School engagement and achievement at time 3 as latent outcomes predicted by ethnic discrimination trajectories: Regression coefficients*

|  | *Outcome:* | **T3 School engagement** | | |  | **T3 School achievement** | | |
| --- | --- | --- | --- | --- | --- | --- | --- | --- |
|  | *Predictors:* | ***B*** | ***(SE)*** | ***β*** |  | ***B*** | ***SE*** | ***β*** |
|  | **Within level** |  |  |  |  |  |  |  |
| *Main effects* | Moderate | 0.02 | (0.09) | 0.04 |  |  |  |  |
|  | High-decreasing | -0.11 | (0.11) | -0.19 |  |  |  |  |
|  | Low-increasing | -0.21^**^ | (0.08) | -0.36 |  |  |  |  |
| *Controls/*  *T1 outcomes* | Age | -0.00 | (0.02) | -0.01 |  |  |  |  |
|  | Gender | -0.07 | (0.06) | -0.11 |  |  |  |  |
|  | School track: Vocational | 0.12^*^ | (0.06) | 0.21 |  |  |  |  |
|  | Parental education | 0.04 | (0.08) | 0.03 |  |  |  |  |
|  | T1 School engagement | 0.62^***^ | (0.08) | 0.60 |  |  |  |  |
|  | T1 School achievement | 0.11 | (0.07) | 0.26 |  |  |  |  |
| *Mediator* | T3 School engagement |  |  |  |  | 3.92^***^ | (1.24) | 0.25 |
| R^2^ |  | 0.38 |  |  |  | 0.14 |  |  |

** p* < .05, ** *p* < .01, *** *p* < .001. *Note.* Standardized estimates are STDY standardization

# Section 10. Latent Outcome Models

We replicated the school non-compliance model at the latent level, to account for the lower values of Cronbach alpha, α = 0.55-0.56, and an engagement model where behavioral disaffection was calculated at the latent level to account for measurement error, as this scale also had lower reliability (α_T1_ = 0.62, α_T3_ = 0.59).

## Latent non-compliance model

Overall, the latent model replicated the findings from the manifested-level non-compliance model presented in the main paper, i.e., following a high-decreasing trajectory was associated with significantly more non-compliance in school at T3, *b* = 0.21, *SE* = 0.10, *p* = .042. The only difference we found was a significant effect of age on T3 non-compliance, suggesting that younger adolescents had more school problems at T3, *b* = -0.08, *SE* = 0.04, *p* = 028. No other within or between regression effects reached statistical significance. The non-compliance latent factor held at the individual level, all standardized loadings were strong (>= 0.44) and significant at *p* < .001 (both standardized and non-standardized). However, at the school (between) level, the non-standardized loadings did not reach statistical significance, suggesting that the scale may not work well at the school level. Overall, the model had an acceptable fit, RMSEA = 0.04, CFI = 0.80, and replicated our findings regarding school non-compliance presented in the main paper.

## Latent behavioral disaffection

We replicated the main findings presented in the manuscript when we defined behavioral disaffection (at T1 and T3) as latent factors (see Table S11). In this model, we kept other measures of school engagement as observed-level variables due to the number of parameters < clusters constraint in multilevel models. In this model, we additionally found a statistically significant effect of ethnic school composition (moderate composition) on lower behavioral disaffection at T3, *p* = .041 (was *p* = .099 in the non-latent engagement model presented in the manuscript). As seen in Table S12, the T1 and T3 behavioral disaffection latent factors held at the individual level, and all standardized loadings were strong (>= 0.44) and significant at *p* < .001 (both standardized and non-standardized). However, at the school (between) level, two of the three non-standardized loadings did not reach statistical significance, suggesting that the scale may not work well at the school level. This model had an acceptable fit, RMSEA = 0.05, CFI = 0.84.

**Table S12** *Engagement model with behavioral disaffection as a latent factor: Standardized factor loadings (with standard errors in parentheses) and intercepts*

|  | T1 Behavioral disaffection | |  | T3 Behavioral disaffection | | | |
| --- | --- | --- | --- | --- | --- | --- | --- |
|  |  | |  | *Within level* | *Between level* | |  |
|  |  | Intercept |  |  |  | Intercept |  |
| I often think of other things during class | 0.63 (0.03) | 2.84 |  | 0.62 (0.05) | 0.99 (0.01) | 43.45 |  |
| In class my thoughts easily wander away | 0.69 (0.04) | 2.69 |  | 0.66 (0.04) | 1.00 (0.00) | 13.45 |  |
| I do not really do my best at school | 0.48 (0.05) | 1.93 |  | 0.44 (0.05) | 0.95 (0.18) | 75.96 |  |

*Note*. All standardized factor loadings significant at *p* < .001.

**Table S11** *Engagement model with behavioral disaffection as a latent factor and ethnic discrimination trajectories as predictors: Regression coefficients*

|  |  | Engagement model | | |
| --- | --- | --- | --- | --- |
|  |  | T3 Behavioral engagement | T3 Behavioral disaffection (latent) | T3 Emotional engagement |
|  | Predictors | ***B (SE)*** | ***B (SE)*** | ***B (SE)*** |
|  | Moderate trajectory | 0.03 (0.09) | 0.20 (0.16) | 0.04 (0.08) |
|  | High-decreasing trajectory | -0.10 (0.11) | 0.53 (0.25)^*^ | -0.02 (0.12) |
|  | Low-increasing trajectory | -0.16 (0.09)^a^ | 0.60 (0.25)^*^ | -0.19 (0.09)^*^ |
| *Controls* | T1 Outcomes | 0.41 (0.04)^***^ | 3.76 (0.50)^***^ | 0.30 (0.04)^***^ |
|  | Age | 0.03 (0.02) | 0.00 (0.05) | 0.03 (0.02) |
|  | School track: vocational | 0.11 (0.06) | -0.15 (0.15) | 0.08 (0.06) |
|  | Gender | -0.05 (0.05) | -0.10 (0.13) | -0.12 (0.05) |
|  | Parental education: higher | 0.05 (0.08) | -0.05 (0.19) | 0.08 (0.06) |
| *R^2^* |  | .18 | .39 | .14 |
| **School-level** | | | | |
|  | Moderate school composition (30-60%) | 0.02 (0.08) | -1.51 (0.74)^*^ | 0.09 (0.10) |
|  | High school composition  (> 60%) | -0.01 (0.08) | -0.75 (0.72) | 0.08 (0.10) |
| *R^2^* |  | 0.45 | 0.31 | 0.94 |

** p* < .05, ** *p* < .01, *** *p* < .001. a *p* =< .81.

# Section 11. Positionality Statement

While reflectivity is a core tenant of qualitative research, it can also be integrated into quantitative research methods (e.g., Jamieson, Govaart & Pownall, 2023). Despite the perceived objectivity of quantitative approaches, biases can permeate various stages, from conceptualization to interpretation (Jamieson et al., 2023). As I am utilizing an existing dataset, I cannot assess how biases may have influenced the selection or formulation of survey items. However, as the corresponding author, my experiences living in the Netherlands and Belgium during post-graduate studies, as someone from a stigmatized Turkish background, might have shaped my perspective. Although not born or raised in these countries, I encountered discrimination in daily life, likely informing my research focus on experiences of ethnic discrimination. It may have also shaped my interpretation that discrimination negatively impacts adjustment. Moreover, I reject oversimplification and the treatment of ethnically minoritized groups as homogeneous, prompting my interest in identifying subgroups experiencing discrimination differently. However, this is a collaborative project in which authors come from different backgrounds and countries, and therefore we also believe that this could help to alter/mitigate any potential implicit bias in our interpretation of the findings.

# References (not cited in the manuscript)

Asparouhov, T. & Muthen, B. (2012). Using Mplus TECH11 and TECH14 to test the number of latent classes. Mplus Web Notes: No. 14. <https://www.statmodel.com/examples/webnotes/webnote14.pdf>

Buhs, E. S., Ladd, G. W., & Herald, S. L. (2006). Peer exclusion and victimization: Processes that mediate the relation between peer group rejection and children’s classroom engagement and achievement. *Journal of Educational Psychology*, 98, 1–13. <https://doi.org/10.1037/0022-0663.98.1.1>

Comrey, A. L., & Lee, H. B. (1992). A first course in factor analysis (2nd ed.). Lawrence Erlbaum Associates, Inc..

Fredricks, J. A., Blumenfeld, P. C., & Paris, A. H. (2004). School engagement: Potential of the concept, state of the evidence. Review of Educational Research, 74, 59–109. <https://doi.org/10.3102/00346543074001059>

Jamieson, M. K., Govaart, G. H., & Pownall, M. (2023). Reflexivity in quantitative research: A rationale and beginner's guide. *Social and Personality Psychology Compass*, 17, e12735. <https://doi.org/10.1111/spc3.12735>

Muthén, L.K. and Muthén, B.O. (2023, September 6). Chi-Square Difference Testing Using the Satorra-Bentler Scaled Chi-Square. Mplus. http://www.statmodel.com/chidiff.shtml

Preacher, K. J., Zyphur, M. J., & Zhang, Z. (2010). A general multilevel SEM framework for assessing multilevel mediation. *Psychological Methods*, 15, 209-233.

Preacher, K. J., Zhang, Z., & Zyphur, M. J. (2011). Alternative methods for assessing mediation in multilevel data: The advantages of multilevel SEM. *Structural Equation Modeling*, 18, 161-182.

Shi, D., Lee, T., & Maydeu-Olivares, A. (2019). Understanding the model size effect on SEM fit indices. *Educational and Psychological Measurement, 79,* 310-334. doi:10.1177/0013164418783530

Stevens, J.P. (1992). Applied multivariate statistics for the social sciences (2nd ed.). Lawrence Erlbaum Associates, Inc.
